# Evidence for Photoinduced Polaron Generation in a High Persistence Length Low Bandgap Conjugated Polymer in Solution

**DOI:** 10.1021/acs.jpcb.5c07501

**Published:** 2026-01-30

**Authors:** Mohammed Azzouzi, Elham Rezasoltani, Matthew J. Bird, Jack F. Coker, Jarvist M. Frost, Garrett S. LeCroy, Anthony W. Parker, Igor V. Sazanovich, Gregory M. Greetham, Michael Towrie, Alise Virbule, Michelle S. Vezie, Despoina Heracleous, Hugo Bronstein, Alberto Salleo, Jenny Nelson, Sophia C. Hayes

**Affiliations:** † Department of Physics, 4615 Imperial College London, London SW7 2BW, U.K.; ‡ Laboratory for Computational Molecular Design (LCMD), Institute of Chemical Sciences and Engineering, 27218 Ecole Polytechnique Federal de Lausanne (EPFL), 1015 Lausanne, Switzerland; § School of Physical and Chemical Sciences, 4617 Queen Mary University London, London E1 4NS, U.K.; ∥ Chemistry Division, 8099 Brookhaven National Laboratory, Upton, New York 11973, United States; ⊥ Department of Chemistry, 4615 Imperial College London, London SW7 2AZ, U.K.; # Department of Materials Science and Engineering, 6429 Stanford University, Stanford, California 94305, United States; ∇ Central Laser Facility, Research Complex at Harwell, STFC Rutherford Appleton Laboratory, Harwell Oxford, Didcot OX11 0QX, U.K.; ○ Department of Chemistry, 54557 University of Cyprus, P. O. Box 20537, Nicosia 1678, Cyprus; ◆ Yusuf Hamied Department of Chemistry, University of Cambridge, Cambridge CB2 1EW, U.K.

## Abstract

Using ultrafast time-resolved infrared (TRIR) spectroscopy, we studied the solution-phase excited-state structural evolution of an indacenodithiophene-*co*-benzothiadiazole polymer (C8-IDTBT). Following band gap excitation, the TRIR spectra reveal vibrational features that develop within 10 ps and decay over 4 ns. Using pulse radiolysis measurements, charge-modulation spectroscopy, and quantum-chemical calculations, the IR features are assigned to polaron pairs. Interestingly, these features appear on an evolving broad mid-IR electronic absorption background, with kinetics correlating with the formation and decay of the cation-radical vibrational bands. A three-state kinetic model successfully reproduces the spectral evolution, revealing that the polaron and exciton populations exist in dynamic equilibrium on picosecond time scales, with time constants for exciton dissociation in the range of 3–5 ps and polaron-to-exciton reformation between 20 and 100 ps, while both species decay to the ground state on much slower nanosecond time scales (∼1 ns), yielding a remarkably high polaron-generation efficiency, higher than 50%. These findings provide fundamental insights into intramolecular charge photogeneration mechanisms in conjugated polymers, demonstrating efficient bound-polaron formation in isolated polymer chains.

## Graphical abstract

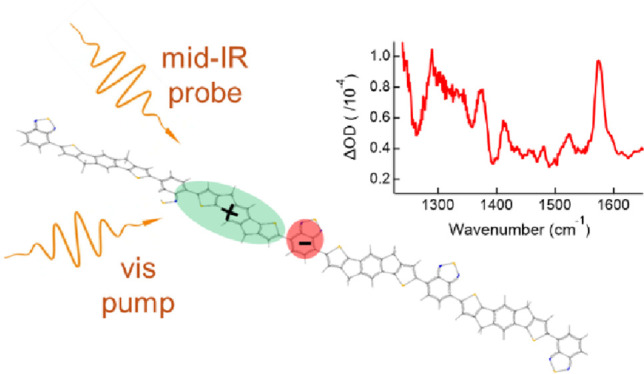

## Introduction

Photoexcitation plays a key role in the operation of organic-based semiconductor devices, especially organic solar cells, and photodetectors. Thus, developing a detailed understanding of the processes that dictate the fate of primary photoexcitation is essential knowledge for the design, optimization, and longevity of such devices. Time-resolved measurements able to follow spectral changes of the system in the visible or near-infrared (IR) region offer insight into the evolution of the excited states involved in the photogeneration process.^1–3^ Although methods such as ultrafast electronic transient absorption have been extensively used to probe the photogeneration process, their use to identify the different species involved as well as correlating spectral changes with structural modifications of the system following the photoexcitation remains challenging due to the spectral overlap of the different species contributing to the induced electronic changes.

Time-resolved mid infrared (TRIR) spectroscopy provides a better molecular probe of the excited states enabling the identification of individual species (e.g., excitons, polarons, triplets) through their unique vibrational signatures, disentangling the time scales of their formation, as well as following excited state relaxation and reorganization (vibrational relaxation, exciton migration, self-localization, conformational changes).^4,5^ Therefore, ultrafast TRIR spectroscopy has been applied to a variety of chemical systems to provide a molecular picture of photoinduced processes, such as charge transfer, charge trapping and charge recombination.^4,6,7^ Furthermore, a number of studies have utilized TRIR to follow the early steps of charge carrier formation in conjugated polymer:fullerene systems, tracking in particular the temporal evolution of the fullerene derivative (phenyl-C61-butyric acid methyl ester, PCBM) carbonyl band (both frequency and intensity), exploiting the sensitivity of this band to the electrostatic field and the dielectric environment.^7,8^ Nitrile and alkyne stretch bands have also been used as vibrational probes of excited state reactions, which, like carbonyl bands, appear in isolated spectral regions.^9–12^ Even though TRIR was used to follow exciton localization in small organic molecules relevant to organic electronics,^13^ only a few studies are found in the literature on probing excited state processes occurring on the conjugated backbone of polymers with TRIR, including some of our work,^14–19^ in contrast to the larger number of studies using femtosecond stimulated Raman spectroscopy (FSRS).^20–27^ An early ps time-resolved photoinduced absorption study by Mizrahi et al. on films of MEH-PPV/C60 blends showed that the photoinduced vibrational features at 300 ps after excitation were the same as the ones observed with quasi-steady-state photoinduced absorption (PIA) assigned to IR-active vibrations (IRAV modes) of the MEH-PPV polaron.^15^ IRAV modes are vibrational modes that become IR-active upon deformation of the polymer backbone due to charge formation.^28,29^ Similarly, TRIR studies by Sakamoto et al. were able to identify the charged species generated upon photoexcitation of PPV, assigned to bound and free polarons.^17^ A TRIR study by Meskers et al. on films of P3HT/fullerene blends with 200 ps resolution showed relatively narrow spectral range IR spectra (1100–1300 cm^–1^) featuring thiophene vibrational modes that were assigned to translational motion of charged excitations along the conjugated backbone,^18^ and these were also correlated to bands observed with quasi-steady state PIA. Furthermore, other ultrafast visible pump, mid-IR probe studies have focused primarily on the generation of free charges tracking the dynamics of the P_1_ polaron electronic band rather than the structural evolution of the polymer backbone.^14,30–32^

Evidence for the IR signature of polarons in conjugated polymer thin films has been widely reported in the literature as mentioned above in the form of IRAV modes observed in quasi-steady state IR absorption spectra from PIA or charge modulation spectroscopy.^33–35^ This polaron signature was observed both in homopolymers and push–pull polymers, with the extent of the overlap with polaron electronic transitions in this region complicating the spectra with the emergence of Fano antiresonances (quantum interference arising due to simultaneous electronic and vibrational absorption).^36–39^ Besides the work of Bakulin et al.,^40^ Stallhofer et al.,^14^ Guo et al.^19^ in polymer thin films and our recent study of a cationic polythiophene in solution where IRAV signatures were observed in the ultrafast TRIR spectra,^16,19^ no other work as far as we are aware reported the generation and evolution of this polaron-assigned IR vibrational signature in conjugated polymers. The accessibility of this mid-IR region with a direct ultrafast structural probe enables disentangling the excited state dynamics following the generation of charged species that is not always evident from near IR TA measurements as we saw in our previous work on a cationic polythiophene, especially in the case of intrachain polarons.^16,41^ Such an accurate and sensitive technique is helpful for determining the nature and role of different excited species in the dissociation of excitons to free charges. The nature of charge photogeneration in organic semiconductors remains one of the most debated topics in the field, with competing models proposing direct exciton dissociation versus formation of intermediate charge-transfer states or polaron pairs.^42–47^ Recent studies have highlighted the importance of hot charge-transfer states and ultrafast charge separation processes that occur faster than vibrational cooling,^48,49^ while others emphasize the role of energetic disorder and morphology in determining dissociation efficiency.^50,51^

In this work, we use ultrafast TRIR in the mid-IR region to follow with structural detail the nature and fate of photoexcitation in a donor–acceptor copolymer. We choose indaceno­[1,2-b:5,6-b′]­dithiophene-*co*-2,1,3-benzothiadiazol with octyl side chains (C8-IDT-BT) as a model polymer system that shows good hole mobility, and reasonable charge generation efficiency when blended with a suitable acceptor molecule.^52,51^ We have previously shown that this polymer has a high persistence length and an associated high extinction coefficient.^53,54^ Moreover, different studies on C16-IDTBT suggest that photoinduced charge generation can occur in these type of polymers.^55,56^ However, the TA work on C16-IDTBT reported in the literature either in solution, films or nanoparticles (NP) suspensions was limited to the near IR region (only down to ∼0.9 eV).^56–58^ In the work by Thomas et al.^56^ on C16-IDTBT solutions and films, any polaron pair formation information had to be extracted from the long-lived species spectra even though the authors alluded to formation on the ultrafast time scales, and this was attributed to polaron pair formation due to interchain interactions. In the work on C16-IDTBT NP suspensions,^57^ the long-lived TA spectra in the near IR showed the existence of triplet excitons rather than polarons, with the latter being enhanced after glycolation of the side chains. Thus, extending the previous work into the mid-IR region where charged species can be directly detected can advance the understanding of the photophysics of this class of polymers. We study C8-IDTBT in solution to follow the dynamics within polymer chains. This offers a direct correlation of photophysics to the backbone structure away from aggregation effects. By combining experimental and computational methods, we deconvoluted the TRIR signal to assign contributions from various species involved in the charge generation process. The use of a kinetic model of the overall process revealed that exciton dissociation into polaron pairs occurs with a 4 ps time constant and a yield higher than 50%, followed by exciton reformation on the tens of ps time scale. Both the exciton and polaron populations persist for a few nanoseconds before ultimately recombining to the ground state through either direct polaron decay or via exciton reformation and subsequent decay pathways. These insights enhance our understanding of exciton dissociation in polymers like IDTBT, which exhibit resilience to disorder and high persistence lengths.

## Experimental Methods

### Materials

C8-IDTBT, synthesized in the McCulloch group (high MW (HMW) *M*_n_ = 459 kDa, PDI = 1.2), was dissolved in deuterated chloroform to a concentration of 0.19 mg/mL (PRU). The low MW (LMW) C8-IDTBT used for comparison had *M*_n_ = 43 kDa, PDI = 3.9. Films of the HMW and LMW polymer were drop-casted on CaF_2_ windows to obtain the FTIR spectrum without solvent interference. FTIR spectra were measured using a Nicolet iS20 instrument (Thermo Scientific).

### TRIR Spectroscopy

The TRIR experiments were performed on the LIFEtime setup at Central Laser Facility.^59^ The setup is built on the dual-amplifier laser system running two 100 kHz Yb:KGW Pharos amplifiers on the same shared oscillator. The amplifier generating the pump beam is rated to produce 15 W average power at 1030 nm at 100 kHz with 260 fs pulse length. The amplifier to generate the probe beam is rated at 6 W average power at 100 kHz and produces 180 fs pulses at 1030 nm.

The excitation beam for the TRIR experiment was set to 670 nm and was generated by the Orpheus HP OPA pumped by the fundamental output beam of the pump amplifier. The probe beam in the mid-IR was generated via a DFG process in GaSe crystals by the two independently tunable OPAs Orpheus-ONE pumped by equally split fundamental beam from the probe amplifier. The probing spectral window presented in this paper from 1200 to 1700 cm^–1^ (5.475–7.1 μm) was covered in the combination of these two probe OPA’s.

The repetition rate of the laser output from each amplifier can be adjusted with a built-in pulse picker. The repetition rate of the excitation pulses was set to 50 kHz. The time delay between the pump and the probe is adjusted with an optical delay line.^59^

The probe beam was overlapped at the sample with the excitation beam, and subsequently the probe beam was spectrally dispersed with a home-built diffraction spectrograph and the spectra recorded with a HgCdTe array detector (Infrared Systems, 128 elements). The resolution of the instrument is 2 cm^–1^. The spot sizes at the sample for the probe and pump beams were ca. 50 and 150 μm (fwhm) and the excitation pump fluence was set to 11 μJ/cm^2^. The samples were presented to the setup in dismantlable IR cells (Harrick Scientific) fitted with 2 mm thick CaF_2_ windows. For the liquid samples, the windows were separated by 250 μm thick PTFE spacers. The film samples were loaded into the same Harrick IR cells. To minimize photodegradation, all samples were rastered in the beam in X and Y directions. To further minimize photodegradation, for the liquid samples the solutions were flown through the cell during experiments.

The TRIR spectra reported are an overlay of at least three spectra from three different spectral regions. The calibration was performed using polystyrene as an external standard.

### Charge-Modulation Spectroscopy

CMS devices were prepared and measured as described previously.^60^ Briefly, polymer thin films (∼50 nm) were spin-coated from a 5 mg mL^–1^ solution of a low molecular weight C16-IDTBT (*M*_n_ = 15 kg/mol, *Đ* = 1.6, synthesized in the Luscombe group; this polymer was used instead due to material shortage for the C8-IDTBT) in anhydrous chloroform onto a 150 nm thick thermally grown SiO_2_ dielectric layer on a double-side polished, lightly doped n-Type silicon substrate (ρ = 1–10 Ω-cm).^61^ The SiO_2_ surface was passivated prior to depositing the polymer layer with a silane layer as follows: a self-assembled monolayer (SAM) was prepared by placing substrates in a 40 mM solution of octadecyltrichlorosilane (OTS) in anhydrous hexanes in a nitrogen-filled glovebox. Substrates were held in the OTS solution for 12 h. SAM-treated substrates were then removed from the hexane solution, moved to ambient atmosphere, sonicated in toluene for 1 min, and then rinsed with isopropanol to remove any OTS residue. These SAM-treated substrates were then annealed in a nitrogen glovebox for 15 min at 130 °C. After spin coating, the polymer films were heated in a nitrogen glovebox at 60 °C for 30 min. Fifteen nm semitransparent gold electrical contacts were thermally evaporated onto the polymer surface, and a 75 nm silver contact was evaporated onto the silicon substrate. Charge modulation spectra were measured by loading the completed CMS devices into a custom nitrogen chamber with KBr infrared transparent windows. Differential device transmission (Δ*T*) was collected by biasing CMS devices to *V*_g_ = −40 V in accumulation and *V*_g_ = +10 V in depletion, where *V*_g_ is the gate bias applied to the silicon substrate. The spectra were collected using a Nicolet iS50R Fourier Transform IR (FT-IR) spectrometer in the spectral range of ν̅ = 450–6000 cm^–1^ (∼1.7–22 μm).

### Pulse Radiolysis

Pulse radiolysis with transient IR absorption (PR-TRIR) was performed at the Laser Electron Accelerator Facility (LEAF) at Brookhaven National Laboratory, as described in detail elsewhere.^62,63^ A ∼7 ns pulse of 9 MeV electrons was used to generate solvent radical cations in deuterated oDCB which then transfer a positive charge to the polymer. ODCB radical anions decay through loss of chloride ions, preventing the formation of IDTBT radical anions. The solution was completely refreshed between each electron pulse due to a buildup of a long-lived product, *P* ^·+^ + *Cl* ^–^.

## Results

C8-IDTBT in deuterated chloroform solution (0.19 mg/mL (per repeat unit, RU), 459 kDa) exhibits two main absorption bands in the visible with maxima at 1.85 eV (670 nm) and 2.95 eV (420 nm) associated with the intramolecular charge transfer and the π to π^★^ transitions, respectively (Figure 1).^56^ Concentration and molecular weight dependent absorption spectra (Figure S1) support that the polymer chains are effectively isolated in the conditions used in this study.

**Figure 1:**
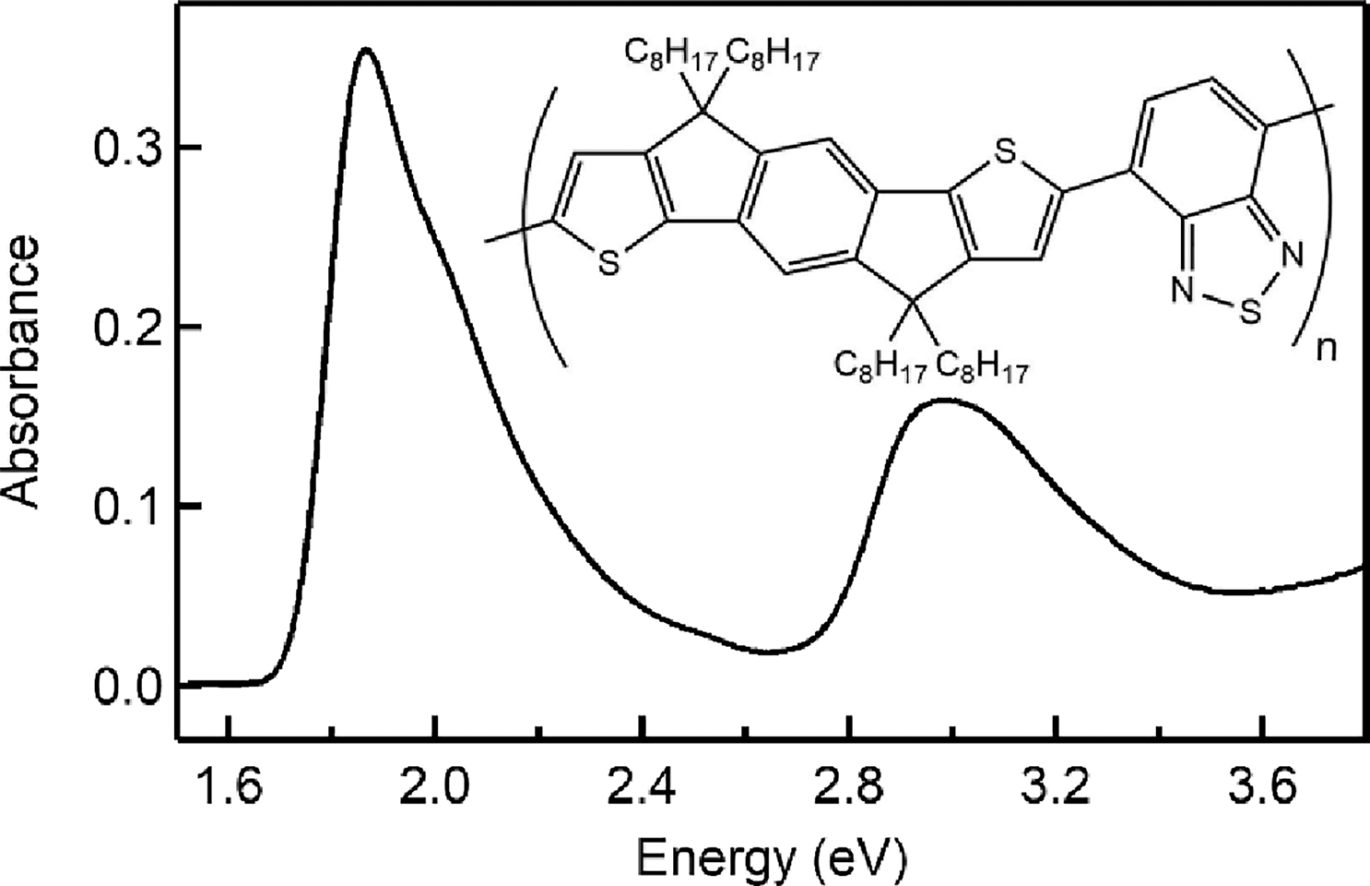
UV–vis absorption spectrum for C8-IDTBT in deuterated chloroform (0.19 mg/mL (per RU), 459 kDa) (Inset: chemical structure of the polymer).

### TRIR Spectra

Excitation within the low energy absorption band (1.85 eV, 670 nm) and probing in the mid-IR spectral region (1300–1700 cm^–1^) produces the TRIR absorption spectra shown in Figure 2a. Similar to electronic transient absorption spectroscopy, positive and negative features are expected following excitation, corresponding to the formation of excited state species and depletion of the ground state, respectively. Additionally, probing in this mid-IR region provides the opportunity to detect any low energy absorption from the excited electronic state (exciton or polaron) to higher lying electronic states, which appears as a broad background under the vibrational features.^6,17,64^ In the TRIR spectra of C8-IDTBT vibrational bands appear within the instrument response (<200 fs) and continue to grow in amplitude for ∼10 ps followed by a slow decay up to 4 ns. In addition, a broad background absorption is evident upon excitation, which then decays with time while evolving spectrally. This broad background is assigned to electronic absorption. We identify the broad spectra associated with the electronic absorption by selecting spectral positions that are not related to a vibrational contribution (i.e., avoiding sharp peaks in the spectra) and then fitting a third-order polynomial function through these points to generate the broad background spectra shown in Figure 2b. We then decouple the electronic and vibrational, contributions to the spectral signature by subtracting the broad baseline spectra from the TRIR spectra at each delay time so that we can track the dynamics of the various features independently (Figure 2b,c). We present more details about the baseline spectra and fitting procedure in the Supporting Information section 2.

**Figure 2:**
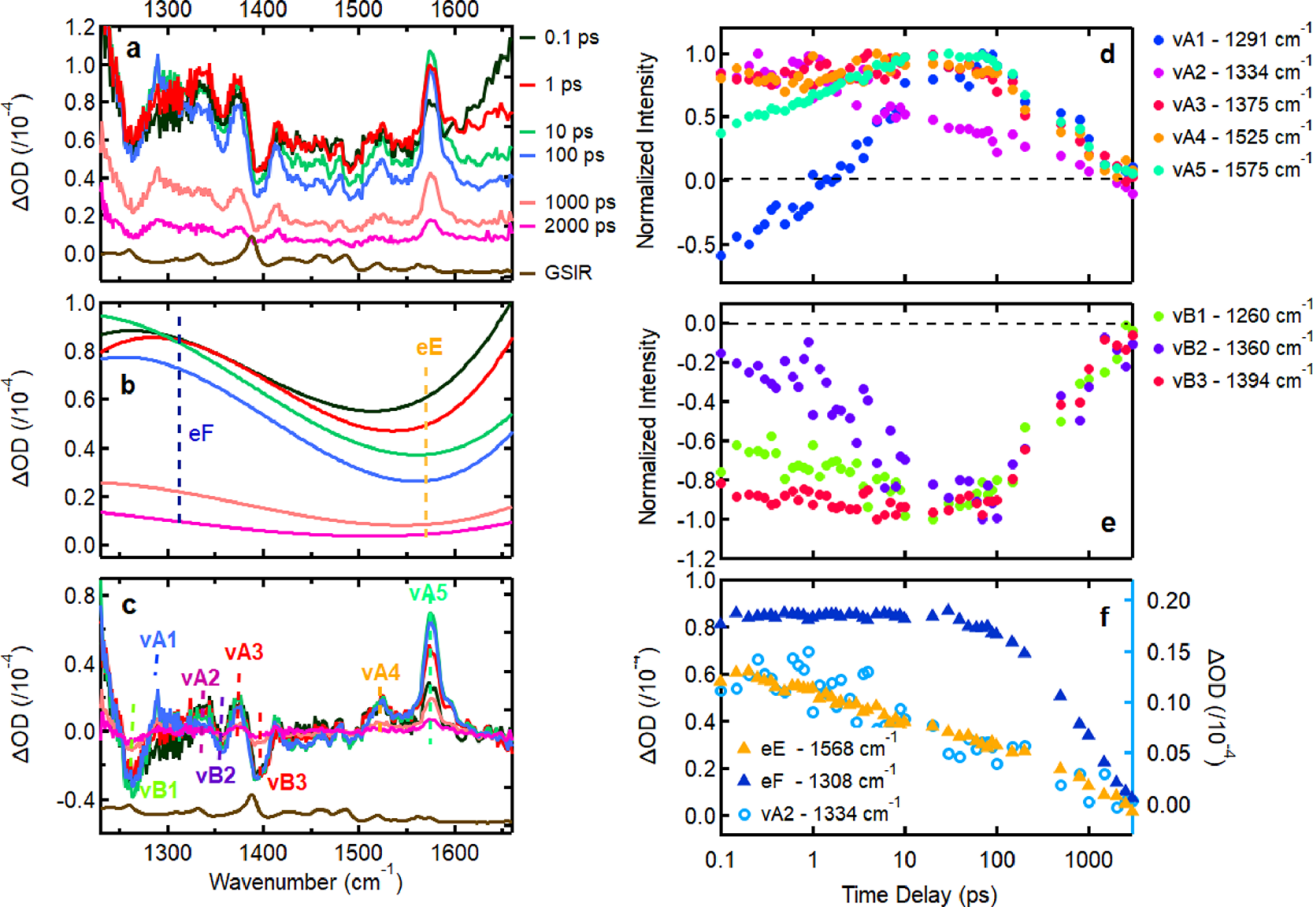
(Left, a) TRIR spectra of C8-IDTBT in deuterated chloroform at select time delays after excitation. (Brown) Measured ground state IR spectrum of drop-cast C8-IDTBT film. (b) Evolution of the broad background absorption spectrum with time as extracted from the TRIR spectra at the same delay times as in (a). (c) TRIR spectra with the background subtracted. (Right) Kinetics of the various vibrational and electronic features of the TRIR spectra at the spectral positions indicated by the dotted lines in (b) and (c): (d) Bands vA1-vA4 due to photogenerated species. (e) Bands vB1-vB3 due to ground state bleach. (f) Electronic absorption baseline kinetics at 1568 (eE, orange) and 1308 cm^–1^ (eF, blue) compared to the evolution of band vA2 (1334 cm^–1^).

The background signal in Figure 2b evolves over time, with the high-frequency component of the spectrum (eE) decaying more rapidly than the low-frequency component (eF). This differential behavior suggests that multiple species may be contributing to the broad absorption feature. Figure 2f presents the kinetics of these two spectral regions. The low-frequency region initially exhibits a plateau, followed by a gradual decay beginning around 10 ps and continuing until approximately 4 ns. In contrast, the high-frequency region displays biphasic kinetics, characterized by a rapid initial decay and a slower decay phase after 10 ps, mirroring the behavior observed in the low-frequency region.

The isolated vibrational spectra shown in Figure 2c display distinct vibrational features that evolve with different kinetics. In total, we identify three dips and five peaks in the 1230–1650 cm^–1^ region. The dips, attributed to ground-state bleach, confirmed by comparison with the ground state IR (GSIR) spectrum of a drop-cast film of C8-IDTBT (see below), occur at 1260 (vibrational Bleach 1, vB1), 1360 (vB2), and 1394 cm^–1^ (vB3). The peaks, associated with excited state absorption, are observed at 1291 (vibrational Absorption 1, vA1), 1334 (vA2), 1375 (vA3), 1525 (vA4), and 1575 cm^–1^ (vA5). Figure 2d illustrates the kinetics of the five vibrational absorption bands, each exhibiting distinct temporal behavior. vA1 (1291 cm^–1^) initially displays a negative dip, that can be possibly attributed to baseline subtraction issues, due to the neighboring vB1 feature at 1260 cm^–1^, followed by a rise up to ∼10 ps, and subsequently decays until 4 ns. In contrast, vA2 (1334 cm^–1^) undergoes a biphasic decay: a rapid component within the first 10 ps, followed by a slower decay extending to 4 ns. Both vA3 (1375 cm^–1^) and vA4 (1525 cm^–1^) exhibit similar dynamics, with an early time signal appearing within the instrument response window, increasing slightly over the first 10 ps, and then gradually decaying through to 4 ns. Finally, vA5 (1575 cm^–1^) appears within the instrument response window, rises continuously over the first 10 ps and then decays similarly to vA3 and vA4 over 4 ns. This different behavior indicates that there is more than one excited species responsible for the set of vibrational features. Similarly, the vibrational bleaches show distinct kinetic behaviors, as illustrated in Figure 2e. vB2 (1360 cm^–1^) exhibits an initial slow rise in the first 4 ps followed by a steady decay of the bleach signal up to 4 ns. In contrast, vB1 (1260 cm^–1^) and vB3 (1394 cm^–1^) display only a rapid increase within the instrument response time, followed by a decay pattern like that of vB2 over 4 ns.

Careful observation of vA5 shows an evolution of the line shape over the experimental time window (Figure 3). At early delay times this band is asymmetric, indicating that multiple peaks contribute to the line shape, while at longer delay times the band becomes more symmetric. Deconvolution of the spectral region between 1560 and 1610 cm^–1^ shows that two peaks (at ∼1572 and 1581 cm^–1^) are sufficient to reproduce the line shape at early times (<4 ps) and only one (at 1575 cm^–1^) at delays longer than 6 ps (Figure S4). This can be explained from the temporal spectral evolution of the contributions of the two peaks; both peaks upshift within the first 3 ps (from 1572 and 1581 cm^–1^ to 1575 and 1585 cm^–1^, respectively), with the contribution of the 1581 cm^–1^ peak diminishing and the 1575 cm^–1^ peak dominating for the rest of the delay times (Figure S5). The shift of the 1581 cm^–1^ peak can explain why a small band at 1596 cm^–1^ is more clearly distinguished at earlier times and broadens on the red side later. Even though the shifts are small, these are outside the error bar of the analysis and greater than the resolution of the instrument (2 cm^–1^) and are reproducible. The position of the 1596 cm^–1^ peak is unaltered throughout.

**Figure 3:**
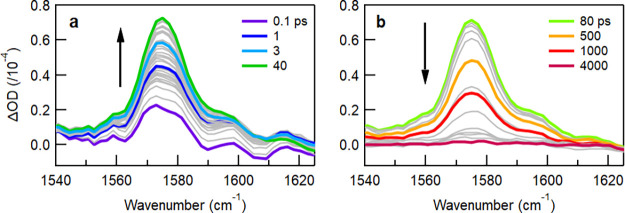
Background-subtracted TRIR spectra of IDTBT in solution zoomed in the spectral region of the prominent vA_5_ band, showing the temporal evolution of the band shape at (a) earlier and (b) later times. Grayed out spectra indicate intermediate delay times with evolution in the direction indicated by the arrows.

### IR Peak Assignment

We next identify and assign the vibrational and electronic spectral features observed in the transient IR spectra and understand their temporal evolution. We use steady state IR spectra of a drop cast film of C8-IDTBT (to avoid solvent contributions that overwhelmed the polymer signal, see Figure S2) to identify the ground state vibrational features and use pulse radiolysis experiments and charge modulation spectroscopy (CMS) to identify the vibrational signature of the polymer cation radical. To help assign vibrational features we use density functional theory (DFT) calculations using the CAM-B3LYP functional and cc-pVDZ basis set to simulate the IR spectra of the IDTBT trimer in its ground state, as well as its photogenerated species: the neutral exciton, cation radical, and anion radical (Figure 4a,b).

**Figure 4:**
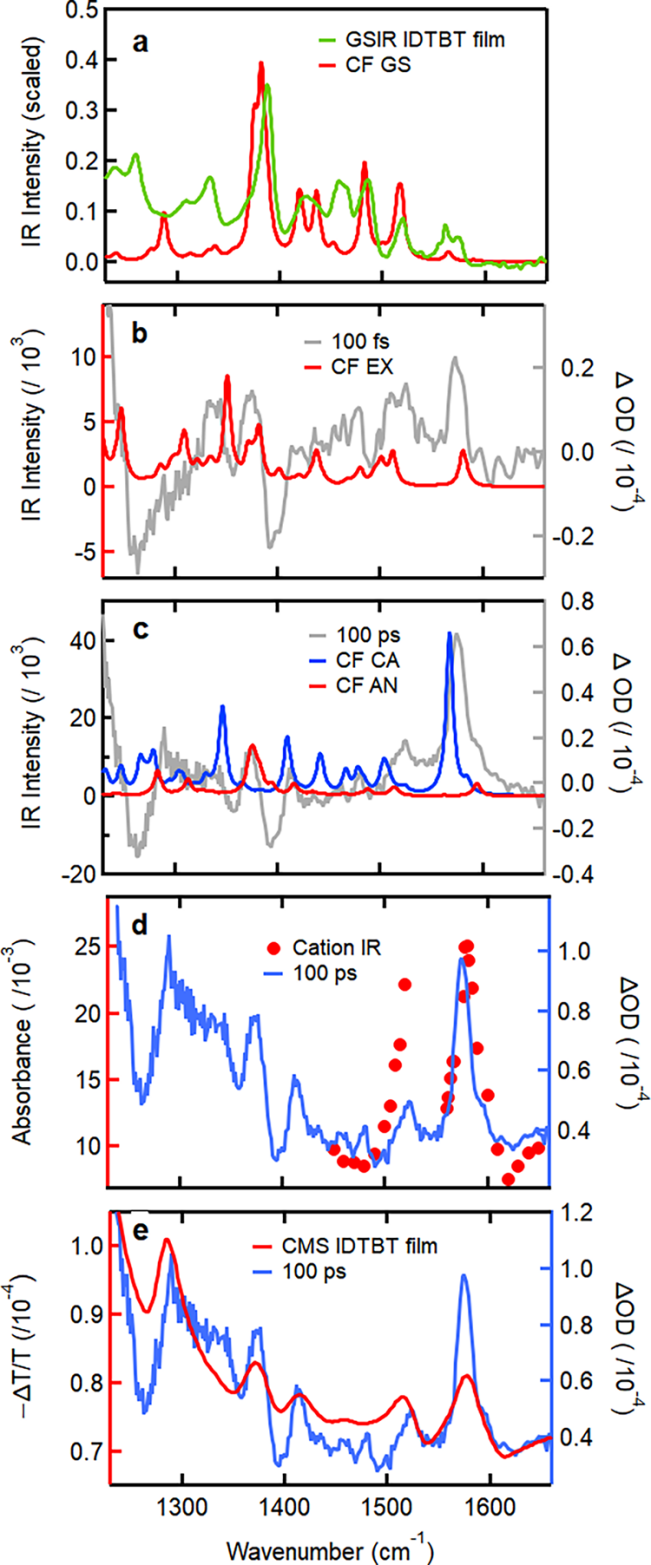
Comparison of (a) the experimental ground state film IR (GSIR) spectrum of C8-IDTBT with computed IR spectrum, (b) the background-subtracted TRIR excited state spectrum of polymer in solution at 100 fs (gray) with calculated exciton (EX) IR spectrum, and (c) the background-subtracted TRIR excited state spectrum at 100 ps (gray) with calculated anion (AN) and cation (CA) radical IR spectra. The calculated spectra were all for an IDTBT trimer in chloroform (CF). The calculated wavenumbers in all cases were scaled by the same factor (0.94), chosen to achieve agreement with the ground state wavenumbers. Comparison of the untreated 100 ps TRIR spectrum (blue) with (d) the radical cation IR spectrum generated through pulse radiolysis (red), and (e) the charge-modulation spectrum for a low-molecular weight C16-IDTBT film (red).

For the ground state features, we find that both the calculated ground-state IR spectrum and the experimental steady state FTIR spectrum show a clear peak at 1390 cm^–1^ (Figure 4a), which corresponds to vB3 in the TRIR spectra (Figure 2c). This band is attributed to the C=C stretch in the thiophene unit of IDT (see Tables S1 and S4). For vB1 and vB2, the experimental FTIR spectrum shows peaks at similar positions, which are reproduced by the calculation of the ground state IR spectrum in chloroform but are of lower intensity than the calculated peak at 1290 cm^–1^. These two peaks correspond to C–H bends either in the BT or IDT unit, respectively, while the 1290 cm^–1^ band corresponds to an asymmetric C=C stretch of the phenyl ring in IDT. Both the calculation and the experimental FTIR spectrum show peaks in the spectral range from 1400 to 1500 cm^–1^ that do not correspond to clear bleaches in the TRIR spectra, which can be due to an overlap with the peaks of photogenerated species, with the overall contributions (negative plus positive) canceling out.

For the assignment of the observed positive TRIR peaks, we consider two types of excited species that could be generated by the photoexcitation: the lowest energy singlet state (S_1_) as we are exciting close to the band edge (1.85 eV), and an excited state with significant electron and hole separation that we will refer to as a polaron pair state (PP). To identify vibrational peaks related to polaron pairs, we compare the TRIR spectra with CMS and pulse radiolysis experiments that directly probe the vibrational and electronic features of radical cations. These experimental techniques provide reliable spectral fingerprints of charged species.^63,65^ We use the radical cation as a proxy for polarons, based on evidence that charge-separated states exhibit similar IR spectra to polarons.^28,37^ We first compare the TRIR spectrum at 100 ps to CMS measurements on C16-IDTBT films[Fn fn1] (Figure 4e). This comparison of different polymers is justified by the fact that the molar extinction coefficient spectra of C8 and C16 IDTBT are almost identical (see SI Figure S6), while the use of film CMS data to interpret solution spectra is justified by the TRIR spectra of both high and low molecular weight films of C8-IDTBT having very similar features to those for solutions (see Figure S7). The CMS and TRIR spectra show strong alignment, especially in the positions of the 1294, 1375, 1513, and 1576 cm^–1^ bands (Figures 4e and S7) and suggest that the vA5 band is strongly related to the radical cation population. The reduced intensity of the 1520 cm^–1^ band in TRIR is likely due to overlapping positive and negative features, as supported by the ground-state IR spectrum, where bleach bands near 1500 cm^–1^ are less intense than the bleach at 1394 cm^–1^. Further validation comes from pulse radiolysis experiments that directly generated C8-IDTBT radical cations via charge transfer from the deuterated ortho-dichlorobenzene (oDCB) solvent radical cation after ionization by a 9 MeV electron pulse. The resulting IR spectrum (Figure 4d) shows a strong peak at 1575 cm^–1^, confirming the role of the radical cation in the vA5 peak. Overlay of the CMS and PR spectra demonstrate the excellent agreement between the radical cation spectra generated with the two different techniques, especially in the relative intensity of the 1513 and 1576 cm^–1^ (vA) bands (Figure S7). Interestingly, the baseline of the pulse radiolysis spectrum between 1600 and 1900 cm^–1^ agrees with the TRIR baseline in this spectral region at later times (Figure S8, Supporting Information), and in conjunction with the calculated IR spectra showing no vibrational bands in this region, we tentatively assign the evolving broad absorption baseline we observe in the TRIR spectra to electronic absorption of a photogenerated radical cation.

The full CMS spectrum seen in Figure S7a indicates that the spectral region probed with the TRIR experiments (1200–1700 cm^–1^, 0.15–0.21 eV) falls in a valley between the low energy bands of the positive polaron electronic absorption of IDTBT (A and B bands according to Spano and co-workers^66,67^ ), explaining thus the absence of Fano antiresonances (seen as bleaches) expected with a better overlap of the electronic absorption band with the IRAV modes, as seen in the C–H stretch region (∼3000 cm^–1^).^68^

These experimental observations are consistent with DFT-calculated IR spectra for the radical cation in chloroform. The simulated spectrum shows strong agreement with several prominent TRIR features (Figure 4c), including band vA5 (1575 cm^–1^), band vA1 (1290 cm^–1^), and the positive band at 1414 cm^–1^ flanking the ground-state bleach band vB3. Another calculated cation absorption band falls on the lower frequency side of vB2 ∼1339 cm^–1^ but does not correspond to a band of appreciable intensity in the TRIR spectrum, possibly due to overlap with the bleach band. These bands are attributed primarily to C=C stretching vibrations across the IDT unit and in some cases (vA1) to C=C stretching in the BT units (see Table S2).

Given the presence of the radical cation, we also considered the possible contribution of the radical anion, especially in the absence of an external electron acceptor. Experimentally, bands at 1373, 1596, and 1523 cm^–1^ in the TRIR spectrum align well with calculated anion modes (in chloroform solvent) at 1375, 1594, and 1515 cm^–1^ (scaled as in the cation case), corresponding to C=C stretches in the IDT phenyl and BT units (Tables S2 and S4). Additional anion-related features may contribute to the bands at 1412 and 1290 cm^–1^, which overlap with cationic and bleach signals.

In order to identify vibrational features due to the first singlet excited state (S_1_) we focus on the early time TRIR spectra and compare with vibrational frequencies calculated using DFT and time-dependent DFT (TD-DFT). We note that isolating the IR spectrum of the singlet in organic molecules or polymers remains a significant experimental challenge due to its transient nature and overlap with ground-state signals. As shown in Figure 4b, we observe a moderate correlation between the calculated and experimental positive peaks of the TRIR spectrum at 100 fs. The TRIR peaks at 1335 (vA2) and 1373 cm^–1^ (vA3) could correspond to the calculated peaks at 1351 and 1383 cm^–1^ (Table S3). The most prominent peak at 1575 cm^–1^ (vA5) in the TRIR spectrum, however, appears upshifted in the calculation, with a small relative intensity as compared to the other peaks. This calculated peak at 1580 cm^–1^ could nevertheless correspond to the lower intensity band at 1581 cm^–1^ revealed after deconvolution (Figure S4). It should be stressed that the intensities of the calculated radical cation IR spectrum are significantly larger (between 5 and 10 times) than the intensities of the exciton bands, in agreement with previous work that reported giant IR intensities for ionic species.^28,69^ Therefore, while it is possible that exciton population also contributes to the TRIR spectra, especially at the earliest times after photoexcitation, the contribution of this state is likely to be overshadowed by the high intensity bands of a charged species.

Having compared experimental TRIR and calculated IR spectra, we acknowledge that simulating IR spectra of conjugated macromolecules in solution using DFT and TD-DFT is challenging due to computational cost and choice of functional. The need to approximate extended polymer chains with explicit solvation by truncated oligomer models or implicit solvent approximations^70^ can affect the accuracy of vibrational frequency predictions, particularly for modes sensitive to chain length or solvent interactions. Additionally, the choice of exchange-correlation functional and basis set plays a critical role in determining the reliability of the calculated spectra. Hybrid functionals such as B3LYP and CAM-B3LYP are commonly used, but they may still introduce systematic shifts in vibrational frequencies or intensities. In the Supporting Information section 5, we investigate the impact of these approximations on the calculated IR spectra of the four species considered above (Ground state, Anion, Cation and S_1_).^71^ Considering the limitations and the results presented in the Supporting Information, we argue that even a moderate agreement between calculated and experimental TRIR spectra is helpful for the assignment of the peaks to a specific electronic state of the molecule.

Now that we have explored the assignments of the vibrational features, we need to rationalize the temporal evolution of the 1575 cm^–1^ band (vA5) that was found to strongly align with the radical cation band in this region. We have described above that we observed an upshift in this IDT C=C stretch in the first 3 ps with a time constant of 0.84 ± 0.25 ps (Figure S5). This could be associated with localization of the polaron. To confirm this hypothesis, we performed DFT calculations of the IR spectra of different size oligomers of IDTBT in the cation state (Figure S9b), wherein we observe the sensitivity of this particular mode to the oligomer length, with an increase in the wavenumber with increased localization. In addition, the diminishing contribution of the 1581 cm^–1^ band after 5 ps, as seen from the deconvolution of this vA5 peak, could be an indication of the dissociation of the S_1_ state to generate the charge separated state (see model below). The calculated exciton band at ∼1350 cm^–1^ and its likely correspondence with the observed TRIR band ∼1334 cm^–1^ could be a second supporting mode for this conversion of the exciton to the polaron state.

Besides the various vibrational features we mentioned above, the evolution of the spectral shape of the baseline absorption (Figure 2b), with the different kinetics at high and low frequencies, suggests that the broad electronic absorption must belong to more than one species. To rationalize the contribution of the different species involved in the charge generation process to this background spectra, we initially calculate the electronic absorption of the first excited state to higher energy states, as well as the electronic absorption of the IDTBT trimer in both radical cation and anion states (Table S5 and Figure S12). Our findings indicate that both excited state absorption and the charged species absorption (cation and anion radicals) significantly contribute to the signal, with overlapping spectra in the region of interest (1300 to 1600 cm^–1^). Considering the decay of eE on two time scales, with the fast one on a similar time scale as the buildup of vibrational bands vA1 and vA3–5 assigned to the radical cation, we can infer that absorption at 1568 cm^–1^ (feature eE) must have contributions from both the exciton and the polaron, with a higher contribution from the former in this region compared to the feature eF at 1308 cm^–1^. Moreover, we find that the broad electronic feature associated with the low frequency region is also present in the CMS data (Figure 4e). This suggests that the cation has a significant electronic absorption in this region.

Based on the comparisons between experimental data and theoretical calculations, we can assign the main TRIR spectral features to specific electronic states; namely the ground state, singlet excited state (S_1_), and polaronic species. The vibrational bleach bands vB1 and vB2 (centered around 1260 and 1360 cm^–1^) align well with the ground-state IR spectrum, but their kinetic profiles suggest additional contributions from excited species (Figure 2e). In contrast, the vB3 band at 1394 cm^–1^ shows strong agreement with both experimental and calculated ground-state spectra and exhibits an approximately monoexponential decay after about 10 ps, indicating it originates solely from the ground state. For the positive absorption features, both the S_1_ and polaron states may contribute. However, bands vA1 (1290 cm^–1^) and vA5 (1575 cm^–1^) show strong correlation with the radical cation spectra from CMS and pulse radiolysis, supporting their assignment to polaronic species. Finally, for the broader electronic features labeled eE and eF, we propose that both S_1_ and polaron states contribute, with varying degrees depending on the spectral region.

### Kinetic Model

To elucidate the population dynamics of the electronic states contributing to the transient IR (TRIR) spectra, we employed a commonly used kinetic model based on a simplified three-state system.^72,73^ Upon photoexcitation, the ground state is depleted, and the singlet excited state (S_1_) is populated. The S_1_ state can either undergo dissociation to form a polaronic species, with a time constant τ_S_1_ → p_, or relax back to the ground state via recombination, with a time constant τ_S_1_ → GS_. Conversely, the polaron state may either recombine to regenerate the S_1_ state (τ_p → S1_) or decay directly to the ground state (τ_p → GS_). We do not consider complete separation of polaron pairs. The analysis script and workflow are available in the repository we have developed for the paper. (https://github.com/mohammedazzouzi15/TRIR_Analysis/).

Analysis of the TRIR spectral kinetics reveals that one species exhibits a rising population within the first few picoseconds. Within the three-state model considered, this behavior is consistent with the formation of polarons from the initially generated S_1_ state. This suggests that τ_S_1_ → p_ operates on a picosecond time scale. The persistence of TRIR signals over several nanoseconds implies that at least one of the ground-state recovery pathways (either from S_1_ or the polaron or both) occurs on a nanosecond time scale. Supporting this, photoluminescence measurements on a structurally similar polymer (C16-IDTBT) in solution show a decay within a few nanoseconds and a quantum yield of approximately 20%.^56^ Given the low oscillator strength of the polaron state, its contribution to photoluminescence is considered negligible. Therefore, the S_1_ population must persist for several hundred picoseconds to nanoseconds, placing constraints on the time constants τ_p → S_1__ and τ_p → GS_.

To reproduce the observed kinetics of the TRIR spectral features, we assume that, in general, each electronic state contributes to the signal at every spectral position, with the magnitude of each contribution governed by its relative absorption cross section. These assignments are informed by the spectral and kinetic analyses described above. Further details of the kinetic model and its implementation are provided in Supporting Information, Section 7.

To constrain the model and improve its robustness, we reduce the number of free parameters. Specifically, we simplify the spectral contributions by reducing the number of free parameters associated with the vibrational peaks and baseline from 28 to 20. Further details on these constraints and their justification are provided in Supporting Information, Section 7.1.

To evaluate how well different parameter sets reproduce the experimental TRIR data, we employ a sequential fitting strategy. First, we choose a set of time constants, then we fit the kinetic traces at the different wavenumbers corresponding to the peaks discussed above. This approach helps mitigate issues related to parameter correlation and ensures a more reliable extraction of kinetic parameters.

Through exploration of the parameter space, we find that reproducing the observed TRIR kinetics requires the exciton dissociation time constant (τ_S_1_ → p_) to lie between 3 and 5 ps and the reformation time constant from polarons (τ_p → S_1__) to lie between 20 and 100 ps. The recombination time constant to the ground state from both the singlet exciton and polaron states must be on the order of 1 ns (Table 1). Among the parameter sets yielding good fits (with chi-square values below 0.7), we observe a notable anticorrelation between the recombination rates of the exciton and polaron states to the ground state, suggesting a compensatory relationship between these pathways in the overall relaxation dynamics. Importantly, the current experimental data cannot definitively distinguish between direct polaron-to-ground state recombination versus polaron-to-exciton reformation followed by exciton decay, as both pathways contribute to the observed signal decay on similar time scales and exhibit this compensatory behavior in the kinetic fitting (Table 1).

**Table 1:** Time Constants for the Various Photophysical Processes as Inferred from the Kinetic Model Fitting to the TRIR Data

**time constant**	**mean value (ps)**	**range of values**
τ_S_1_ → p_	4.42 ± 1.25	3 to 6 ps
τ_p → S_1__	72 ± 47	10 to 100 ps
τ_S_1_ → GS_	1100 ± 1400	0.6 to 4 ns
τ_p → GS_	1270 ± 1301	0.1 to 5 ns

Figure 5 presents the results of the best-fit model. In panel 5b, the reproduced kinetics of the various vibrational peaks are shown, demonstrating that the model successfully captures the distinct temporal behaviors associated with each peak. We also show in panel 5b the baseline kinetics for eE and eF, which are also well-represented by the model, further validating its accuracy across different spectral features. In panel 5c, we plot the evolution of the population of the different electronic species considered, where we see the transfer of population from the exciton to the polaron pair in the first 4 ps. Then the populations of these two species decay with a similar rate to the ground state. Almost 80% of the excitons dissociate to form polarons.

**Figure 5:**
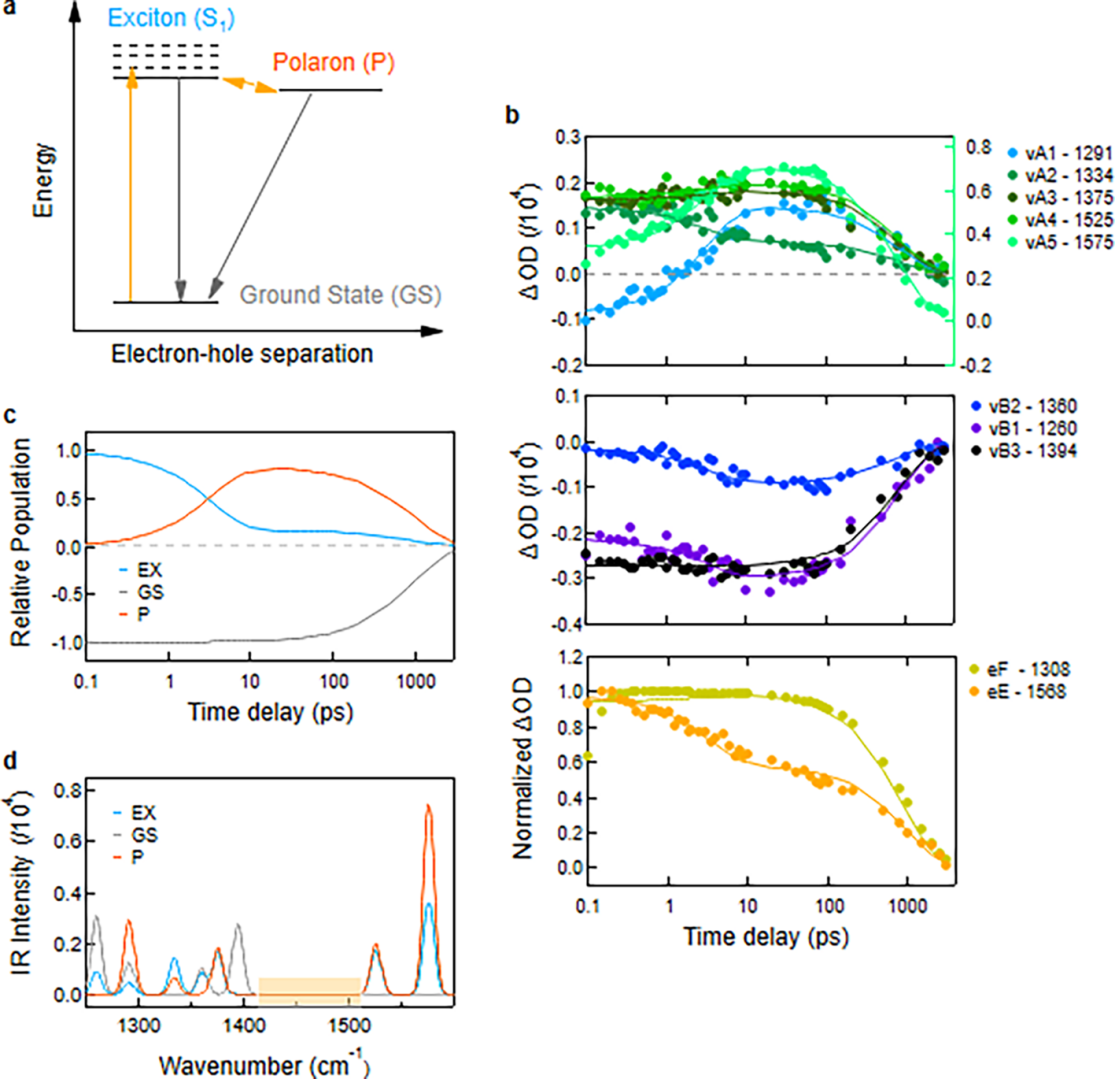
(a) Schematic representation of the processes leading to the polaron formation used in the kinetic model. (b) Fit to the kinetic traces of the different vA and vB peaks identified in the TRIR spectra (top and middle panel), as well as the low and high frequency region of the baseline spectra (bottom panel). (c) Relative population kinetics of the three states used in the model. (d) Inferred IR spectra for the different electronic species. These spectra are computed from the different absorption cross sections inferred by fitting the three-state model to the data, with a broadening of each peak using a Gaussian function with sigma of 5 cm^–1^. Note: the inferred spectra only reproduce the contributions of the various species at the spectral positions analyzed (shaded orange box: spectral region not considered in the analysis). The model results shown in this figure are with the following time constants: τ_S_1_,GS_ = 3 ns, τ_S_1_,P_= 4 ps and τ_P,S_1__ = 20 ps.

We infer IR absorption spectra associated with each electronic state using the relative cross sections derived from the kinetic model (Figure 5d). To simulate the spectral features, we convolve the contributions of each state at each wavenumber with a Gaussian function of 5 cm^–1^ width. The resulting vibrational peak intensities and positions show good agreement with previously measured ground-state IR spectra, CMS data, and theoretical calculations. It should be stressed that only the highlighted peaks considered in the fits above (vA1–5 and vB1–3) are reproduced in the inferred spectra, and not the entire spectra as in the DFT calculations, to avoid errors due to the small signal in this region. For the ground state, four distinct peaks are well reproduced, both in terms of position and relative intensity. For the polaron state, all four characteristic peaks are present in the CMS spectra, with relative intensities closely matching those inferred from the model. In contrast, the S_1_ state shows partial agreement with calculated spectra, with some vibrational features captured but moderate discrepancies in relative intensities, likely due to limitations in the computational model or spectral overlap.

From the kinetic model, the estimated polaron generation yield, which represents the maximum fraction of photoexcited species converted to polarons, reaches more than 50% (the yield distribution is shown in Figure S16), a considerably high value for this class of materials. While the extracted parameters are robust, such a yield prompts further scrutiny of the model’s assumptions. In the absence of evidence for additional contributing states, we turn to independent experimental data to validate the absorption cross sections and refine the yield estimate. The CMS spectra provide a reliable basis for this analysis, as the charge density in the accumulation layer is precisely controlled by the dielectric capacitance and applied voltage. By comparing the intensity of the 1575 cm^–1^ polaron band in the 10 ps TRIR spectrum—where it reaches its maximum—with the ground-state bleach band at 1387 cm^–1^, for which we also determined a molar extinction coefficient (see Supporting Information, Section 8), we calculate a polaron quantum yield of 40 to 80%. Furthermore, independently from the calculated absorption cross sections, an estimation of the quantum yield can be made based on the extent of the ground state bleach in the TRIR spectra right after excitation and at 10 ps delay time, yielding a value of ∼80%, which is in line with the value from CMS. The experimentally determined quantum yield supports the model’s predictions and confirms that polaron formation is significantly high in this system.

To further validate these findings, we measured the TRIR response of a C8-IDTBT film (deposition details in the method section) to complement the dilute solution measurements. While film analysis presents additional complexities due to intermolecular interactions and alternative decay pathways, applying the same analytical approach yields consistent results. As detailed in Section 7.3 of the Supporting Information, we observe a similarly high yield of bound polarons after 5 ps, followed by recombination on a nanosecond time scale. These film measurements corroborate the solution-phase findings, confirming that efficient polaron formation is an intrinsic property of the C8-IDTBT polymer chain rather than an artifact of the solution environment.

## Discussion

From the experimental results and their extensive analysis along with the model, two key insights emerged.

### Population Dynamics

The time-resolved evolution of the electronic state populations, shown in Figure 5b, reveals a substantial yield of polaron formation, estimated to be higher than 50%. These values are high for a polymer in solution. Such yields are not always calculated or explicitly stated, and when they are, they tend to be much lower. For example, transient absorption (TA) studies on regioregular P3HT (RR-P3HT) films reported yields of only 6%,^1^ while more recent TA/FSRS measurements suggest a 14% yield.^27^ In donor–acceptor (D–A) polymer films, Tautz et al. observed with TA yields up to 24%, compared to just 8% in homopolymers.^74^ Kahmann et al. noted remarkably high polaron yields in neat push–pull polymer films using mid-IR detection, although they did not quantify these values.^75^ Moreover, examples of photoinduced charge separation within single polymer^76^ and molecule^46,77^ domains in thin films are known, where charge separation is believed to be intermolecular. However, our findings concern a dilute polymer sample where intramolecular charge separation must be considered. A previous THz conductivity study of various conjugated polymers in sufficiently dilute solution has indicated a prominent peak conductivity in C16-IDTBT compared to the other polymers studied, which they attributed to possibly a high initial charge yield (though not quantified) or a pronounced initial charge mobility. A high initial charge yield would agree with our findings.^55^

The exceptionally high polaron generation yield observed in our spectroscopic analysis (higher than 50%) suggests a highly efficient initial charge separation process, which is a critical prerequisite for effective photocurrent generation in organic photovoltaic (OPV) devices. In principle, such yields should correlate with strong device performance, as polaron formation is a prerequisite for photocurrent generation. Despite IDTBT’s excellent intrinsic charge mobility and low energetic disorder,^78^ the charge generation in single component solar cells using this polymer remains low.^52,79^

The rapid reformation of excitons from polarons can act as a bottleneck for efficient charge generation. Although polarons are initially formed with high yield, a fast extraction rate is essential to prevent their reformation into excitons or relaxation to the ground state. A simple calculation allows us to estimate the polaron extraction rate in the solid state, assuming a charge carrier mobility of approximately 0.1 cm^2^ V^–1^ s^–1^ and a polaron size of around 10 nm.^80^ If we completely neglect any interaction between polarons prior to full separation, under typical solar cell operating conditions and with a device thickness of 100 nm, this corresponds to an extraction rate on the order of 1–10 ns^–1^. Applying this rate within our model yields a charge generation efficiency estimate above 20%. However, the presence of Coulombic attraction between even well separated polarons would prevent separation via drift at any field typically encountered in a solar cell. More details about the impact of the extraction rate and the calculation of the estimate can be found in the Supporting Information (Section 7.2). Further investigation could help to clarify the factors limiting polaron extraction in practice, and more comprehensive measurements of polaron formation kinetics across different OPV polymers are necessary.

### Thermodynamic Implications

The extracted time constants of exciton dissociation and polaron-to-exciton reformation can be used to estimate the free energy difference between the two states.^72^ Considering that the system must obey detailed balance, the ratio of forward and reverse rate constants relates to the difference in energy between the two states and the ratio of state densities (degeneracy of the state) through a Boltzmann factor:

$$\frac{\tau_{p\to S1}}{\tau_{S1\to p}}=\frac{g_{S1}}{g_{p}}e^{-\Delta G_{S1,p}/k_{B}T}$$ 1

where Δ*G*_S1,p_ is the free energy between the S_1_ and polaron, *k*_B_ is Boltzmann constant and *T* is the temperature. *g*_S1_ and *g*_p_ are the degeneracies of the S_1_ and polaron states, respectively. Hence, we can infer from the time constants considered that the polaron state is 20 to 100 meV lower in energy than the S_1_. Figure S16 shows the distribution of the difference in energy between the polaron and the S_1_ state considering the distribution of time constants that fit the experimental results.

The suggestion that the polaron pair state lies below the lowest energy singlet state is surprising, since exciton states in organic materials are strongly stabilized by Coulomb interactions and are expected to lie well below a charge-separated state. We searched for evidence of an excited state with high electron hole separation with energy close to the lowest energy singlet by calculating the properties of the excited states using time-dependent (TD)-DFT (See Supporting Information section 9 for more details). Specifically, we looked at the degree of charge transfer (CT) character of low-lying states in different systems, including: (i) Hexamers of C8-IDTBT in the ground state geometries (Supporting Information section 9.1.1); (ii) Hexamers in geometries extracted from molecular dynamics simulations of single chains immersed in solution (Supporting Information section 9.1.2); (iii) Hexamers and trimers in both ground state optimized geometry and first excited state optimized geometry (Supporting Information section 9.1.3); (iv) Trimers of C8-IDTBT in different solvents and considering different functional and basis set (Supporting Information section 9.1.4); (v) Different pairs of contacting oligomers (pairs of dimers) with geometries extracted from molecular dynamics simulations of molecular aggregates in the solid state (SI section 9.2). Following this thorough investigation, we only see clear evidence for the presence of a low energy excited state with a strong CT character when considering intermolecular systems (case (v) with the B3LYP functional (Figure S30). None of the other calculations unequivocally indicate the presence of a low energy excited state with strong CT character that could enable exciton dissociation. Furthermore, invoking the excited states of contacting oligomers to explain the appearance of polaron pairs is unsatisfactory for several reasons. One is that since the experiment is done in dilute solution, the intermolecular charge transfer pathway is unlikely to dominate. Second, the results for the interacting dimers show a strong exchange-correlation functional dependence, with the lowest energy excited state demonstrating CT character when employing the B3LYP functional, but not when employing the CAM-B3LYP functional (Figure S31). The absence of CT character in the CAM-B3LYP calculations suggests that the low-lying CT state predicted by B3LYP may be a numerical artifact resulting from insufficient long-range Hartree–Fock exchange. Moreover, TD-DFT is limited in its ability to accurately capture the degree of CT character of excited states. This does not mean such states are nonexistent, especially given the experimental evidence provided here that a state with charge-transfer character is involved in the excited state evolution; rather, it highlights the need for more advanced methods capable of accurately modeling these phenomena. To fully investigate the charge generation process in these systems, one option would be to simulate the nonadiabatic excited-state dynamics following photoexcitation in a sufficiently long oligomer, which could capture different possible state relaxation processes.^81^ However, considering the size of the molecular system that would need to be simulated, these types of calculations are unfeasible using state-of-the-art methods and are hence currently out of the scope of this work. This discrepancy underscores the power of our experimental approach to capture essential state evolutions that state-of-the-art theory cannot yet resolve, posing a provocative challenge for future studies to bridge the gap between observation and simulation.

## Conclusions

In this work, we have used ultrafast transient Infrared (TRIR) spectroscopy to probe the evolution of photogenerated excitations in C8-IDTBT polymer in dilute solution. Probing the evolution of the signal in the mid-IR region gives a convoluted signature with contribution from different vibrational features of the different species involved in the charge generation process, as well as a broad background related to their electronic transitions. We have successfully applied a combination of different experimental and computational methods to distinguish the contribution of the different species to the spectra. We used pulse radiolysis, charge modulation and steady-state IR spectra of the different species to identify the position of the vibrational peaks. With supporting DFT calculations we were able to compare the relative strength of the experimental signals of the different species and assign their contributions to the observed spectra, and TD-DFT to assess the contribution of the different species to the background electronic absorption. Overall, we presented a methodology for deconvoluting TRIR signals to extract useful information.

Using all the information above, we built a kinetic model to explain the charge generation dynamics stemming from photoexcitation of C8-IDTBT. We conclude that the exciton dissociates into a polaron pair (an electronic state with spatially separated electron and hole polarons) in this highly diluted system with a time constant of 3–5 ps and a yield higher than 50%. Moreover, we find that the polaron pair signal persists for several nanoseconds because the primary recombination pathway is via reformation and subsequent decay of the exciton. The model results suggest that the polaron pair state should be slightly lower in energy than the excited state and should have strong charge transfer character. Examples of photoinduced charge separation within single polymer^76^ and molecule^46,77^ domains in thin films are known, where charge separation is believed to be intermolecular. However, our findings concern a dilute polymer sample where intramolecular charge separation must be considered. Although prior results on similar polymers suggest that the polaron formation may be facilitated by the ease of polaron delocalization and intrachain charge transport along the IDTBT backbone,^65,78^ excited state calculations using TD-DFT did not yield evidence for a low-lying excited state with strong charge transfer character. This leaves the mechanism behind the photogeneration process open to interpretation. We anticipate that a computational method capable of resolving the dynamics of excited states would be better equipped to explain why such a high yield of polaron formation is observed in this system. In addition, systematic studies of different polymer systems both in solution as a function of solvent polarity and in thin film using combined time-resolved vibrational and electronic spectroscopy will help establish a unified theoretical framework for exciton dissociation processes and resolve ongoing debates.

## Supplementary Material



## Footnotes

^1^
The measurement was not available for the C8-IDTBT due to material shortage.

## Notes

Additional details on spectral analysis, comparison of TRIR spectra with PR and CMS results, calculation of the polaron quantum yield, computational methods and detailed results (vibrational mode assignments, electronic absorption spectra), description of the kinetic models considered, and calculations searching for the low energy CT state (PDF)
