# Supplementary material for: Evidence for Photoinduced Polaron Generation in a High Persistence Length Low Bandgap Conjugated Polymer in Solution
Source: J Phys Chem B. 2026 Jan 30;130(6):1991–2004. doi: 10.1021/acs.jpcb.5c07501 (PMC12908121; doi:10.1021/acs.jpcb.5c07501)
Supplement: Supplementary file 1 [file jp5c07501_si_001.pdf]

Supporting information

Evidence for Photoinduced Polaron Generation in a High Persistence Length Low Bandgap Conjugated Polymer in Solution

Mohammed Azzouzi,[1,2] Elham Rezasoltani,[1,3] Matthew J. Bird,[4] Jack F. Coker,[1] Jarvist M. Frost,[5] Garrett S. LeCroy,[6] Anthony W. Parker,[7] Igor V. Sazanovich,7 Gregory M. Greetham,[7] Michael Towrie,[7] Alise Virbule,[1] Michelle S. Vezie,[1] Despoina Heracleous,[8] Hugo Bronstein, [5,9] Alberto Salleo,[6] Jenny Nelson,*[,1] Sophia C. Hayes*[,8]

[1] Department of Physics, Imperial College London, SW7 2BW, London, UK

[2] *Laboratory for Computational Molecular Design (LCMD), Institute of Chemical Sciences and Engineering, Ecole Polytechnique Federal de Lausanne (EPFL), 1015 Lausanne, Switzerland*

[3] School of Physical and Chemical Sciences, Queen Mary University London, E1 4NS, London, UK

[4] Chemistry Division, Brookhaven National Laboratory, Upton, New York, 11973, United States

[5] Department of Chemistry, Imperial College London, London SW7 2AZ, London, UK

[6] Department of Materials Science and Engineering, Stanford University, Stanford, CA 94305, United States

[7]Central Laser Facility, Research Complex at Harwell, STFC Rutherford Appleton Laboratory, Harwell Oxford, Didcot, OX11 0QX, UK

[8]Department of Chemistry, Univ. of Cyprus, P.O. Box 20537, Nicosia, 1678, Cyprus

[9] Yusuf Hamied Department of Chemistry, University of Cambridge, Cambridge CB2 1EW, UK

Contents

1. Steady-State Spectroscopy of C8-IDTBT 3
2. Baseline subtraction from raw time-resolved IR spectra 4
3. Charge Modulation Spectroscopy of IDTBT films 7
4. Pulse Radiolysis Experiments 8
5. DFT and TDDFT calculations 8
   5.1. Calculation of IR spectrum 8
      5.1.1. Impact of the oligomer size 8
      5.1.2. Impact of the solvent 9
      5.1.3. Impact of the DFT functional 11
   5.2. Assignment of experimental vibrational bands from calculated spectra 12
6. Excited state absorption spectra. 18
7. Model for charge generation 20
   7.1. Reproducing the experimental data 21
   7.2. Charge extraction efficiency 26
   7.3. TRIR data for C8-IDTBT film. 28
8. Calculation of the polaron quantum yield 31
9. Nature of the excited state: looking for the low energy CT state. 34
   9.1. Intramolecular Excited States. 34
      9.1.1. Ground state geometry: 34
      9.1.2. MD generated geometries. 36
      9.1.3. Impact of excited state relaxation 37
      9.1.4. Impact of the solvent and the basis set and functional 38
   9.2. Intermolecular Excited States 40
      9.2.1. Impact of Basis set and Functional 42
      9.2.2. Impact of Solvent 44
10. References 45

1. Steady-State Spectroscopy of C8-IDTBT

Highly dilute solution (5 µg/ml) UV-vis spectra of the C8-IDTBT polymer at different molecular weights (Mn ranging from 43 - 459 kDa) in o-DCB (Figure S1) show insensitivity to the MW, either as far as spectral position or shape and transition strength. The absorption spectrum of the 459 kDa polymer featured in this study at the higher concentration used (0.19 mg/ml per repeat unit (RU)) has a similar shape with the 43 kDa polymer used at similar concentrations, and with the more dilute solution spectra. If aggregation was occurring, then the shape and/or strength of absorption would change with MW, as the higher MW may be expected to aggregate. Furthermore, comparison of the drop-cast film spectra to the solution spectra of the corresponding MW polymers (43 and 459 kDa) used in this study shows that the film spectra are red-shifted (~12 nm) and their shape is modified with a more pronounced side-band shoulder as expected for interacting polymers. Therefore, we assume that at the concentrations we use in deuterated chloroform the polymer chains are non-interacting.

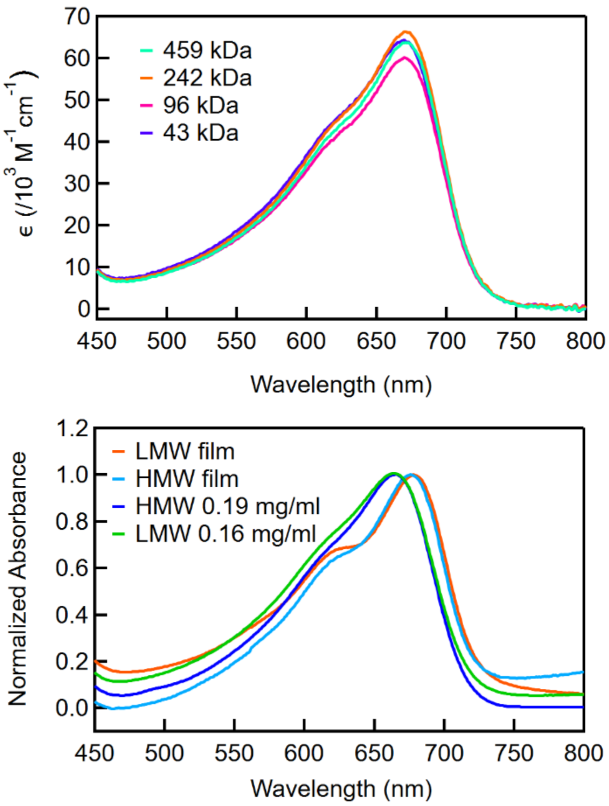

Figure S1: (Top) Extinction coefficient spectra for various molecular weight C8-IDTBT fragments in dilute solution (5 µg/ml in 1,2-dichlorobenzene). The extinction coefficient values are per repeat unit. (Bottom) Comparison of absorption spectra for HMW (459 kDa) and LMW (43 kDa) C8-IDTBT polymers in deuterated chloroform solution or drop-casted film.

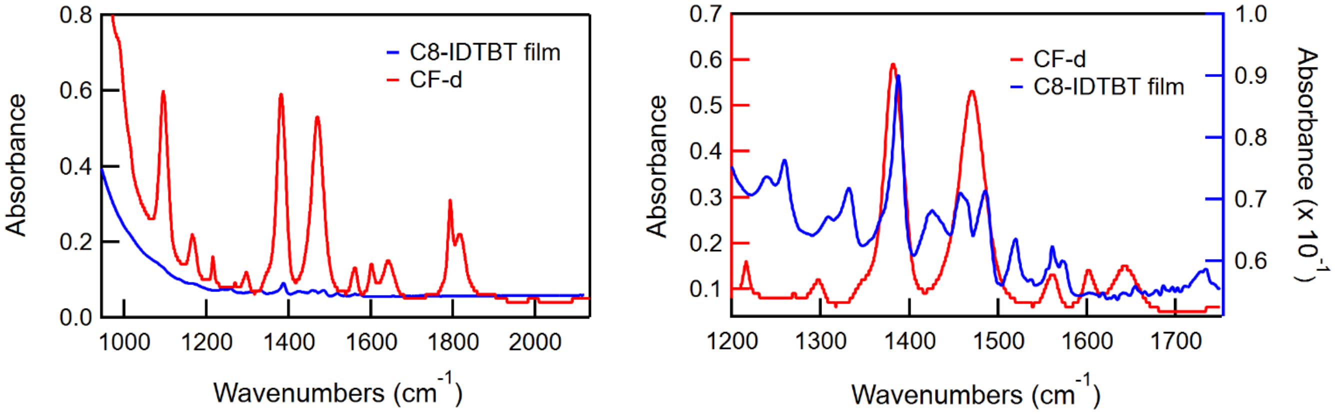

Figure S2. Comparison of the FTIR signal from the solvent (chloroform-d, CF-d, 250 µm pathlength) and the drop cast film of C8-IDTBT. (Left) In scale and (right) overlaid using two different axes to see more clearly the spectral overlap that necessitates comparing the TRIR spectra with a film of the polymer.

2. Baseline subtraction from raw time-resolved IR spectra

For the subtraction of the baseline from the raw TRIR spectra, spectral positions with no contribution from vibrational sharp features were selected and were used as anchor points for simultaneously fitting all the spectra at all time delays. A 3rd order polynomial function was fitted through these points and was used as the baseline for each spectrum as seen in Figure S1. Subsequently, these polynomial fits were subtracted from the corresponding TRIR spectra to generate background-free vibrational spectra, in order to more easily follow the structural evolution of the system.

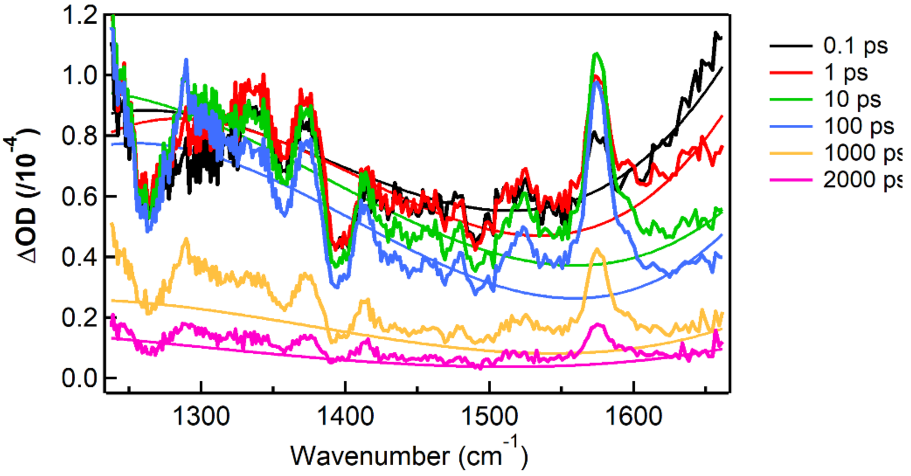

Figure S3: Time resolved IR spectra prior to background subtraction. Superimposed are the baseline fits for each delay time based on a third order polynomial.

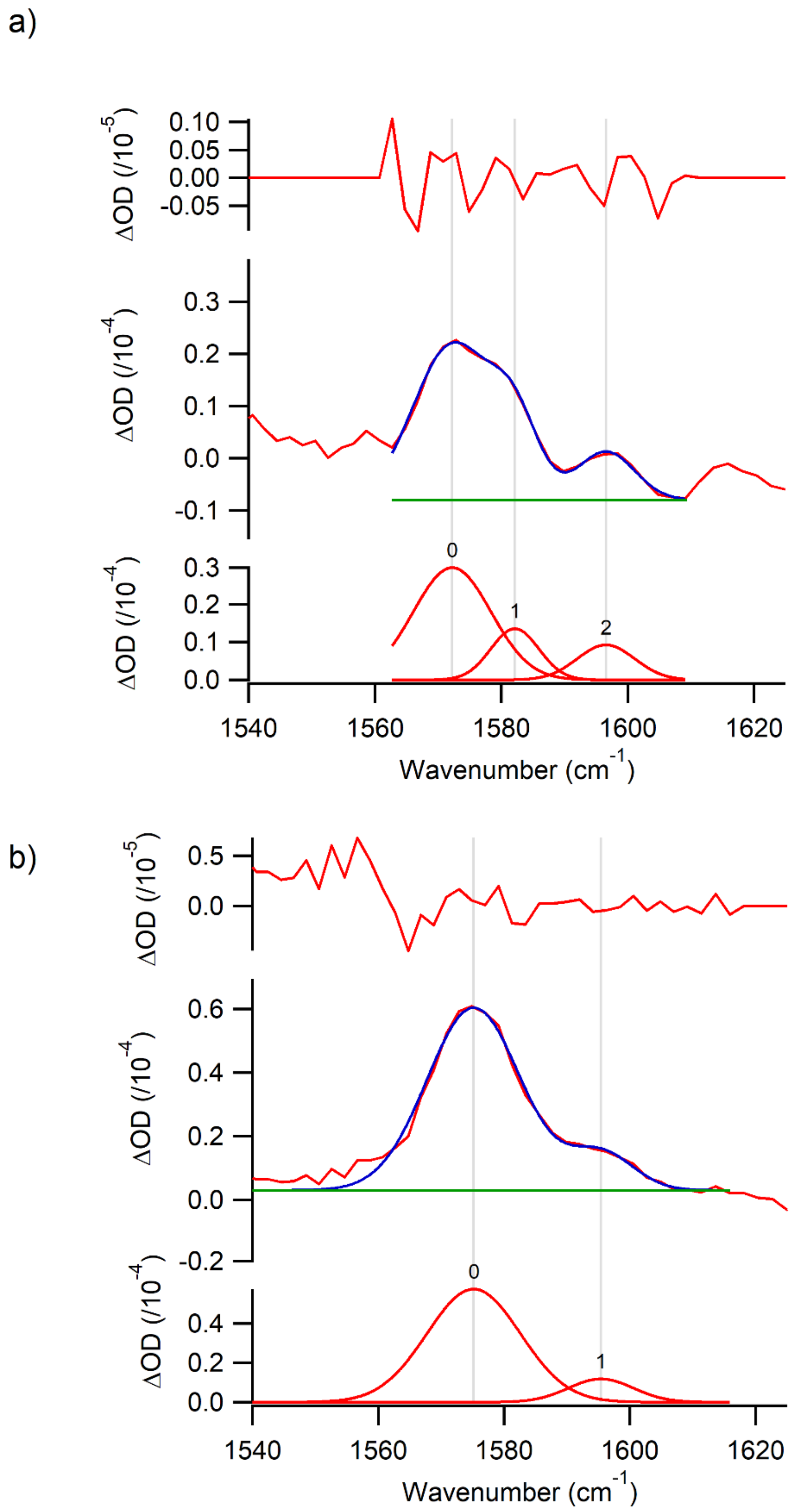

Figure S4: Band deconvolution of the TRIR spectra at a) 100 fs and b) 200 ps. In each panel, (bottom) individual peaks used to fit the cation and anion bands, (middle) overall fit to these bands, (top) residual of the fit.

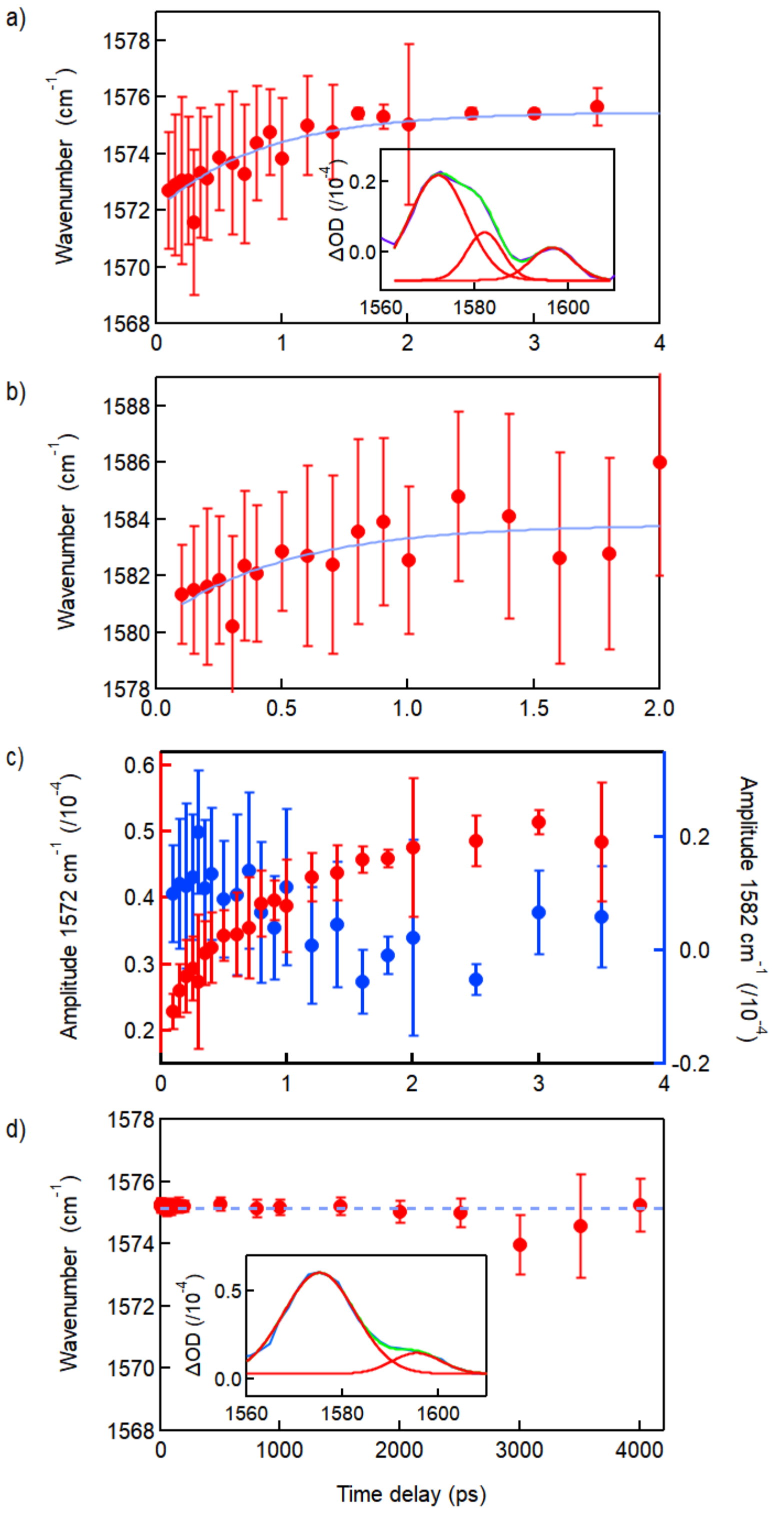

Figure S5: Kinetics of the frequency shift of the band at ~1574 cm^{-1}. The kinetics in (a) and (b) resulted from the fit to this band using two peaks as in Fig. S3(a) for delay times up to 4 ps, while in (d) from the fit using one peak as in Fig. S3(b) for delay times from 6 ps until 4 ns. The kinetics in (a) and (b) are fit to a mono-exponential function: (a) $y = y_0 + A \times e^{-\frac{x - x_0}{\tau}}$, with y_0 =1575.5 ± 0.3, A = -3.0877 ± 0.335, x_0 = 0.1, and τ = 0.84 ± 0.25, and (b): y_0 =1583.8 ± 0.6, A = -2.8364 ± 0.816, x_0 = 0.1. (c) Temporal evolution of the amplitudes of the 1572 (red) and 1582 cm^{-1} (blue) bands, showing the anticorrelated contribution to the 1574 cm^{-1} band.

3. Charge Modulation Spectroscopy of IDTBT films

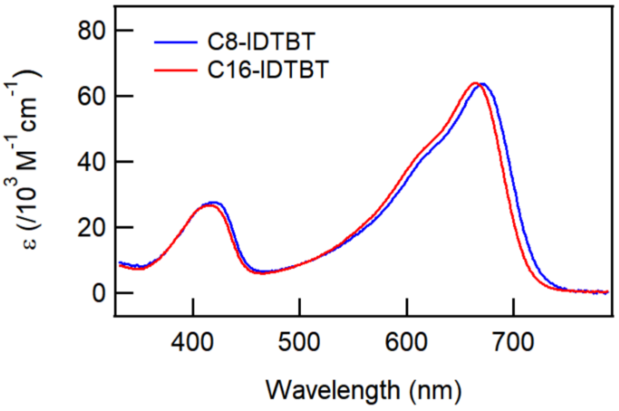

Figure S6: Decadic extinction coefficients per monomer of C8-IDTBT and C16-IDTBT polymers measured in dilute solution (5 µg ml^{-1} in 1,2-dichlorobenzene).

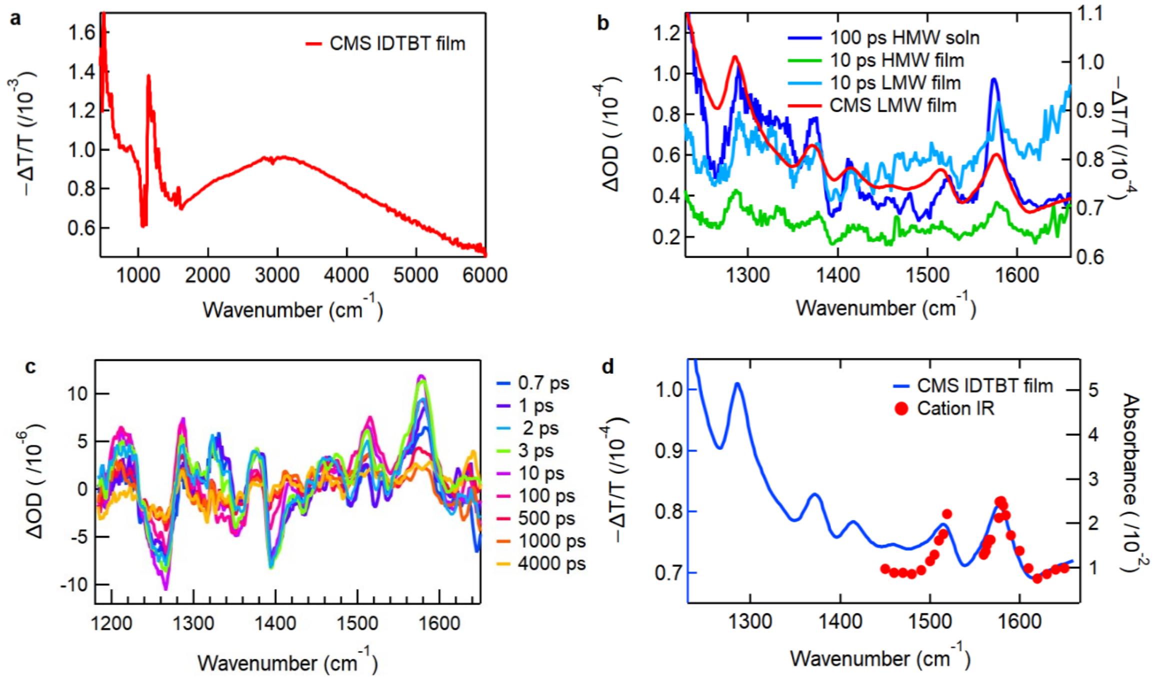

Figure S7: (a) Full charge modulation spectrum for a C16-IDTBT LMW film (15 kDa). The sharp dip at ~1100 cm^{-1} is thought to be an artifact of stark effect shifts in the absorption from the 150 nm SiO_2 gate dielectric used in CMS samples. The dashed box indicates the spectral region of our TRIR experiments. (b) TRIR spectra of C8-IDTBT films for HMW (459 kDa) and LMW (43 kDa) polymer and comparison to the CMS spectrum and the TRIR spectrum of HMW C8-IDTBT in solution. (c) Background-subtracted TRIR spectra of HMW C8-IDTBT film. (d) Comparison between CMS (C16-IDTBT, blue) and PR (C8-IDTBT, red) spectra.

4. Pulse Radiolysis Experimentss

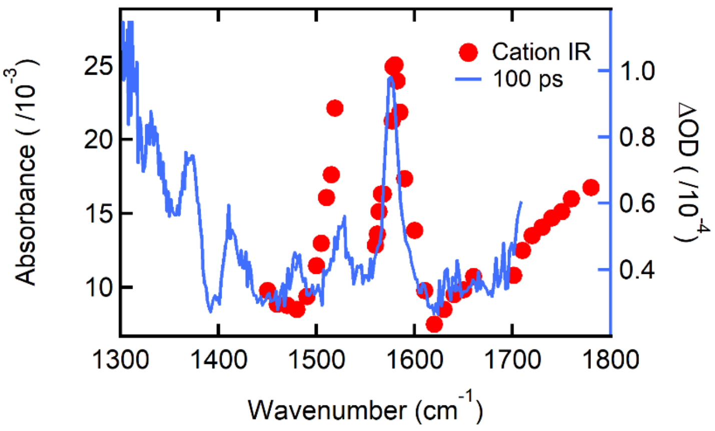

Figure S8: Comparison of the TRIR spectrum at 100 ps delay time with the full pulse radiolysis spectrum, in order to compare the absorption baseline at higher frequencies.

5. DFT and TDDFT calculations

5.1. Calculation of IR spectrum

For the DFT and TDDFT calculations we used the CAM-B3LYP exchange-correlation functional with the cc-pVDZ basis. We calculated the vibrational absorption intensities and their frequencies using Gaussian 16. For the Ground state, Cation and Anion calculation, we first optimized the geometry of the trimer and then calculated the IR absorption spectra. For the excited state, we used TDDFT to optimize the geometry of the trimer in the first excited state and then perform the frequency calculation from it.[1]

In this section we explore the impact of the different approximations on the calculated IR spectra. Here we consider the impact of: 1) size of the oligomer considered; 2) Impact of the polarised medium (solvent); 3) Impact of the functional used for the calculation.

5.1.1. Impact of the oligomer size

Here we show the impact of changing the size for the oligomer considered in the calculations. Here the calculations are all done in Vaccum and using CAM-B3LYP exchange-correlation functional with the cc-pVDZ basis set.

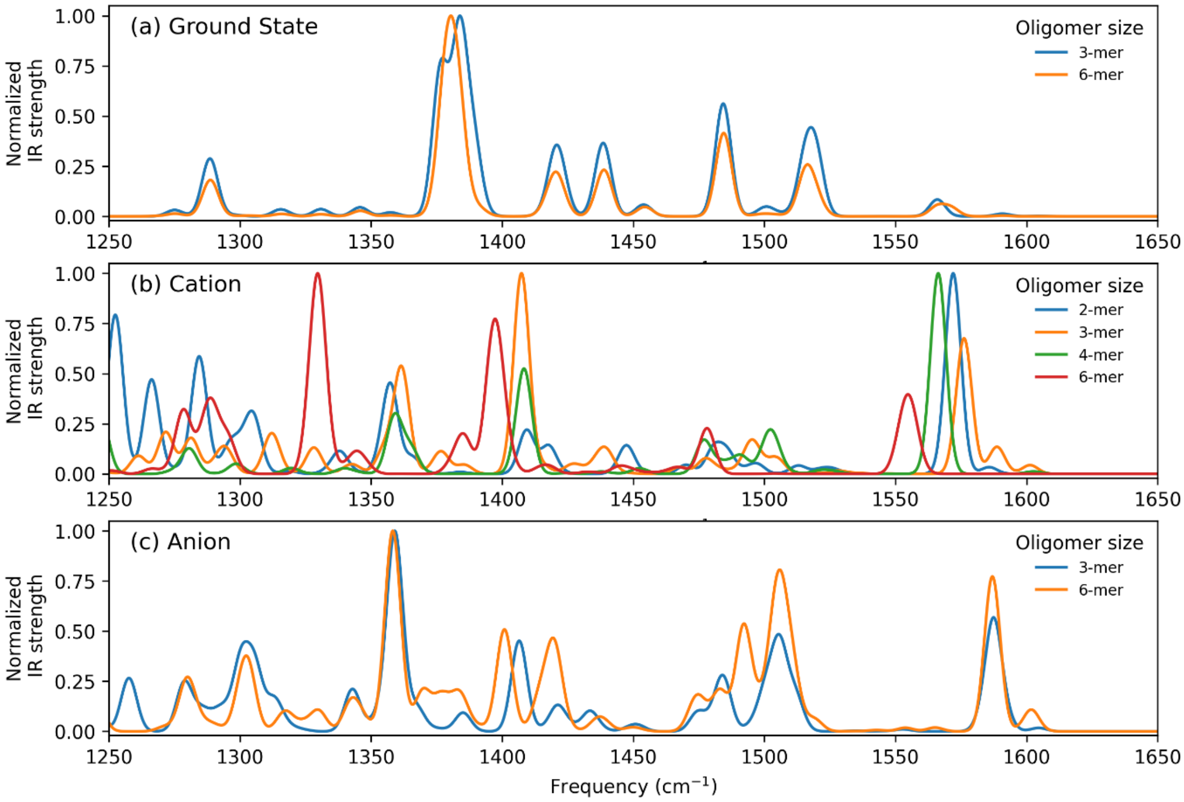

Figure S9: Oligomer size effects on IR spectra of IDTBT oligomers. Normalized IR spectra (1250-1650 cm^{-1}) for IDTBT oligomers of different sizes in S_0, cationic (CA), and anionic (AN) states. All the frequencies are scaled with 0.94 to reproduce the experimental spectra. These calculations are for the single molecules in vacuum.

Figure S8 shows the calculation results of the normalised IR spectra in the region (1250-1650 cm^{-1}). For the three states (ground state, CA and AN) we consider the two sizes of the oligomer (3-mer and 6-mer). The calculation for the ground state and Anion show limited change of the spectra with oligomer size. The relative intensity of the peaks is slightly affected, and some extra peaks are present in the 6-mer as compared to the 3-mer in the case of the AN. For the cation, we observe a bigger impact of the oligomer size on the spectra. Specifically, we see a strong shift of the peak around 1570 cm^{-1}, which is close to the strong vA5 peak in the TRIR spectra.

5.1.2. Impact of the solvent

Here we show the impact of considering different solvents for the calculation. We consider the solvent as a polarized continuous medium, modeled using its dielectric constant. The solvents considered (water (ε ≈ 78.4), chloroform (ε ≈ 4.81), and vacuum (ε = 1.0)) have significantly different dielectric properties, which influence the electronic structure

calculations. All calculations are performed for the trimer using the CAM-B3LYP exchange-correlation functional with the cc-pVDZ basis set.

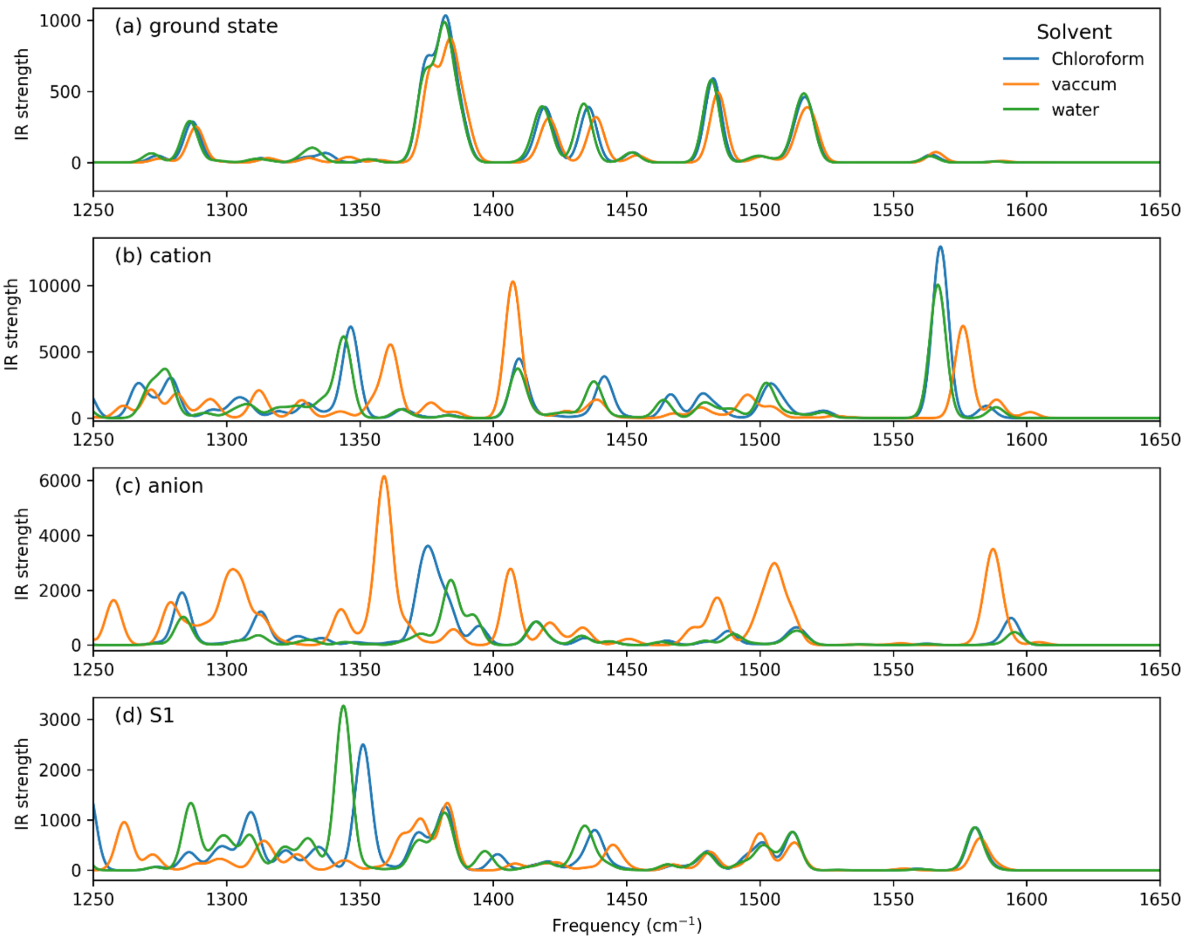

Figure S10: Solvent effects on IR spectra of 3-IDTBT. IR spectra (1250-1650 cm^{-1}) in S0, cationic (Ca), anionic (An) states, and S1 state. All the frequencies are scaled with 0.94 to reproduce the experimental spectra

Figure S9 shows the calculation results of the IR spectra in the region (1250-1650 cm^{-1}) for the 4 states (GS, CA, AN and S1) in three different solvents. For the ground state, the solvent has very small impact on the spectra, which is expected as the state is not polarized. For the Cation, the impact of the solvent is more prominent, showing a strong downshift of the peak at 1575 cm^{-1} and the peak at 1350 cm^{-1}. Similarly, the Anion shows a strong upshift with the solvent. For the S1 state, the introduction of a polarised medium results in the appearance of a strong peak at 1350 cm^{-1}.

5.1.3. Impact of the DFT functional

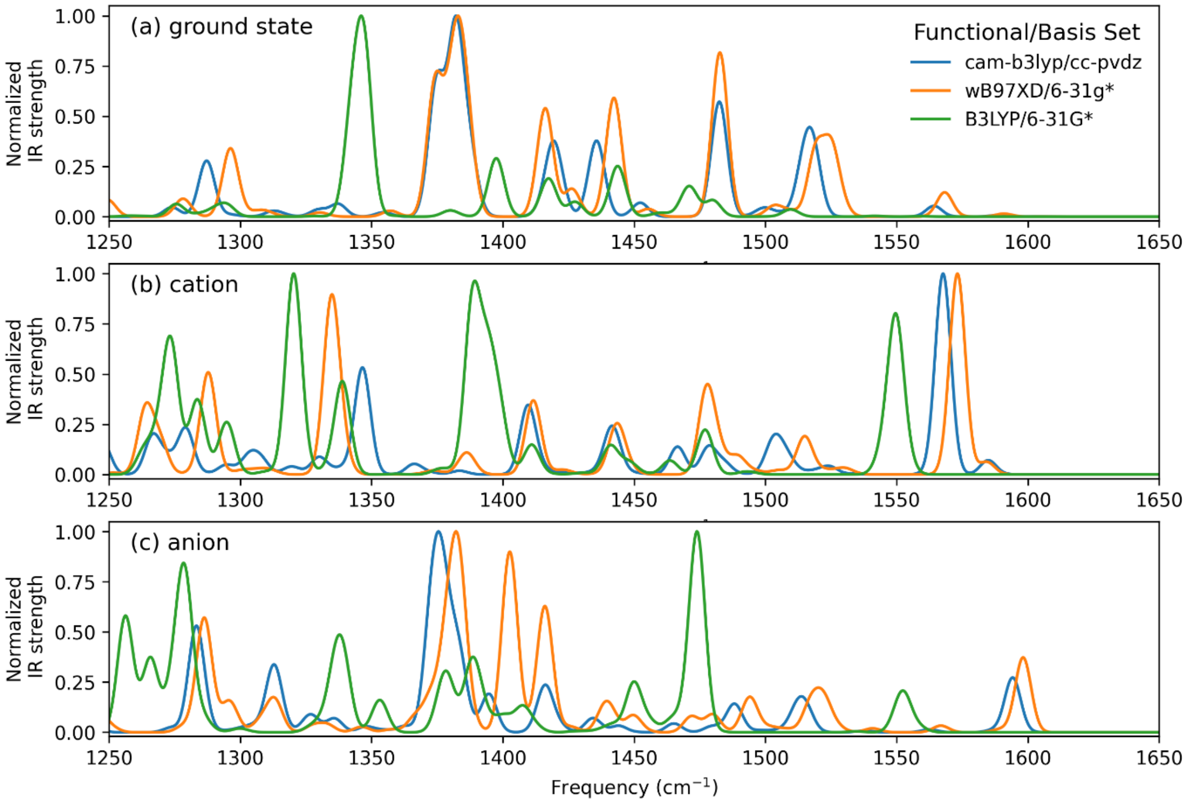

Figure S11: Functional and basis set effects on IR spectra of 3-IDTBT. IR spectra (1250-1650 cm^{-1}) for IDTBT oligomers in S0, cationic (Ca), anionic (An) states, and S1 state. All the frequencies are scaled with 0.94 to reproduce the experimental spectra

We investigated the sensitivity of calculated IR spectra to the choice of density functional and basis set for the ground state, cation, and anion species (Figure S10). Three different functionals were compared: B3LYP (a hybrid functional without long-range correction), CAM-B3LYP (a range-separated hybrid functional), and ωB97XD (a range-separated functional with empirical dispersion). For the ground state, CAM-B3LYP and ωB97XD show excellent agreement in both peak positions and relative intensities, while B3LYP exhibits significant frequency shifts that are inconsistent with experimental IR spectra. This trend is also observed for the cationic and anionic states, where the two range-separated functionals (CAM-B3LYP and ωB97XD) demonstrate good overall agreement, though some differences in peak intensities are noted. These results highlight the importance of incorporating long-range exchange interactions for accurate prediction of vibrational frequencies in charged and neutral IDTBT systems.

5.2. Assignment of experimental vibrational bands from calculated spectra

The tables below include the assignments of the vibrational features observed in the ground state FTIR spectrum of a drop cast film of C8-IDTBT (459 kDa) (Table S1) and the TRIR spectra of the C8-IDTBT solution in chloroform (Tables S2 and S3). Table S2 assigns the contributions of cation and anion radical species to the TRIR spectra, while Table S3 contains assignments to the singlet exciton. For clarity, it is important to note that the various species that contribute to the TRIR spectra (i.e. cation/anion radical, singlet exciton) have overlapping contributions in some cases, which is the reason for multiple assignments to an experimental band as seen e.g. in Table 2. The contribution of the various species to the TRIR spectra has been reproduced by our kinetic model that gave the inferred spectra shown in Figure 5 of the main text. In addition, in some cases the observed bands are broad and include transitions from multiple calculated bands (e.g. Table S1 and S3).

Table S 1 Assignment of GS IR bands from DFT calculations in vacuum.

**Exp. $\bar{\nu}$** (cm^{-1})	**GS IR** Calc. $\bar{\nu}$ (cm^{-1})[1]		**Description[2]**
	3IDTBT_ D10_GS	3IDTBT_ D180_GS	
1240	1290	1284	δ(C-H) + ring def. (IDT) / ρ(C-H) (BT)
1260	1318		ρ(C-H) (BT)
	1371	1371	ν_{as}(C=C) + δ(C-H) phenyl r + ρ(CH_2) on each CP (IDT)
1309	1399		ν_{as}(C-C) (CP, IDT)
1331	1415	1395	ν(C=C) between phenyl and thiadiazole
1387	1464<br>1472<br>1478	1465	ν_s(C=C) (T, IDT)
1428	1510	1503	ν_s(C-C) (T, IDT)
	1530	1531	δ(C-H) phenyl r (IDT)
1460	1547	1550	ν(C-C) either side of phenyl r (IDT) – oop
1486	1579	1578	ν_{as}(C-N) (BT)
1520	1612<br>1616	1609<br>1618	ν_{as}(C=C) (T, IDT)<br>ν_s(C=C) (BT)
1561	1666	1677	ν_{as}(C=C) (BT)
1574	1692	1696	ν_{as}(C=C) (BT)

[1]Unscaled wavenumbers.
[2]Notation: ν – stretch, δ – bend, ρ – rock, def – deformation, s – symmetric, as – asymmetric, oop – out-of-phase, T – thiophene, IDT – indacenodithiophene, BT – benzothiadazole.

Table S 2 Assignment of transient IR bands from cation and anion calculations in chloroform.

**TRIR** (exp) (cm^{-1})	**Cation IR** Calc. $\bar{\nu}$ (cm^{-1})[1]	**Description**[2]	**Anion IR** Calc. $\bar{\nu}$ (cm^{-1})	**Description**[2]
1215[3]	1256	ρ(C-H) (BT) + δ(C-H) phenyl r/T (IDT)	1289	δ(C-H) + phenyl r def. (IDT)
			1299	ρ(C-H) (BT)
	1327	ρ(C-H) (BT) + δ(C-H) phenyl r/T (IDT)	1323	ρ(C-H) + ν_s(C-N) (BT)
	1348	δ(C-H) + ring def + ν(C-N) (BT) + ν(C-C) between BT and T (IDT)		
1289	1361	δ(C-H) + ring def (BT)	1365	ρ(C-H) + r def (IDT / BT)
	1386	ρ(C-H) + ν_{as}(C=C) phenyl (IDT) + ρ(C-H) + ν(C=C) (BT)	1396	ν(C=C) (CP, IDT)
	1414	δ(C-H) (CP)		
1335		---		----
	1432	δ(C-H) (CP) + ν(C=C) between T and CP, inter-ring ν(C-C) IDT and BT +r def (BT)		----
	1453	ν(C=C) (T, IDT) + ν(C=C) (BT)		----
1373		--	1464	ν(C=C) (T, IDT) + out-of-phase ν(C-C) between BT and IDT
		--	1484	ν_{as}(C-N) + ν_{as}(C=C) / ρ(C-H) (BT) + ν(C-C) T next to BT
1412	1500	ν(C=C) (T, IDT) + ν(C=C) + δ(C-H) (BT)	1507	ν(C-C) (T, IDT) + ρ(C-H)/ ν(C-N) (BT)
1435	1560	ν(C=C) + δ(C-H) (BT) + ν(C-C) + δ(C-H) T on either side		
1479	1572	ν_{as}(C=C) / ν(C-C) on different IDT T's + ν_{as}(C=C) + δ(C-H) (BT)	1583	ν_{as}(C=C) T + δ (C-H) (T, IDT)

1512 (sh)	1600	ν_{as}(C=C) + δ(C-H) (BT) + ν(C-C) (T, IDT)		----
1523		----	1612	ν(C=C) (BT) + δ(C-H) (BT)
1574	1668	ν_s(C=C) / δ(C-H) phenyl (IDT)		---
1596			1696	ν_s(C=C) / δ(C-H) phenyl (IDT)

[1]Unscaled wavenumbers.
[2]Notation: ν – stretch, δ – bend, ρ – rock, def – deformation, s – symmetric, as – asymmetric, sh – shoulder, T – thiophene, IDT – indacenodithiophene, BT – benzothiadazole, CP - cyclopentane, T - thiophene. [3]from film spectra of C8-IDTBT (see Figure S8).

Table S 3 Assignment of transient IR bands from calculation of the S_1 state.

**TRIR** (exp) (cm^{-1})	**S_1 IR** Calc. $\bar{\nu}$ (cm^{-1})[1]	**Description[2]**
1215[3]	1291	ρ(C-H) (BT) + δ(C-H)/ r breathing (T, IDT)
	1327	δ(C-H) + ring def (BT)
1289	1393	ν(C=C) + δ(C-H) (BT) + r def (IDT)
1335	1437	ν(C=C) between T and CP + δ(C-H) (CP)
1373	1458	ν_s(C=C) (T, IDT) and +r def (BT)
	1470	inter-ring ν(C-C) T and BT + ν(C=C) (T, IDT)
1412		
1435	1530	ν(C=C) + δ(C-H) (IDT)
1479	1575	ν_{as}(C-N) + ρ(C-H) (BT) + ρ(C-H) + r def (T, IDT)
1512 (sh)	1597	ν_{as}(C=C) + δ(C-H) (T, IDT) + ρ(C-H) + r def (BT)
	1609	δ(C-H) + ν(C=C) (BT)
1581	1681	ν_s(C=C) / δ(C-H) phenyl (IDT)

[1]Unscaled wavenumbers.
[2]Notation: ν – stretch, δ – bend, ρ – rock, def – deformation, s – symmetric, as – asymmetric, sh – shoulder, T – thiophene, IDT – indacenodithiophene, BT – benzothiadazole, CP - cyclopentane, T - thiophene. [3]from film spectra of C8-IDTBT (see Figure S8).

Table S4 Images of most important vibrational normal modes in the ground state, cation and anion IR spectra.

**GS IR**<br>Expt. $\bar{\nu}$ (Calc. $\bar{\nu}$, vacuum)
1560 cm^{-1} (1666 cm^{-1})<br>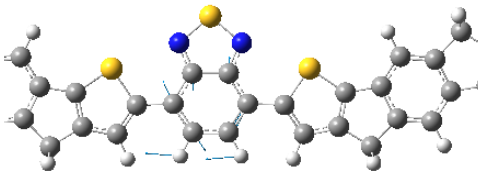
1520 cm^{-1} (1612 cm^{-1})<br>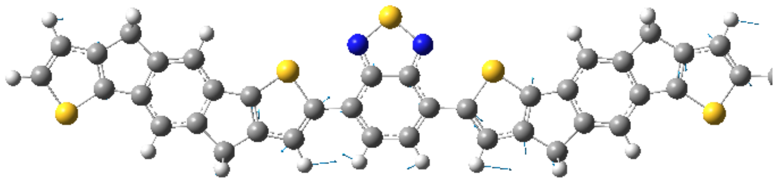
1486 cm^{-1} (1579 cm^{-1})<br>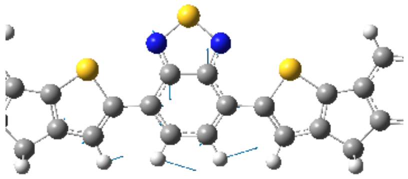
1387 cm^{-1} (1472 cm^{-1})<br>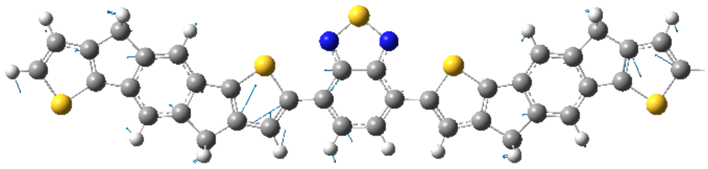
1260 cm^{-1} (1371 cm^{-1})<br>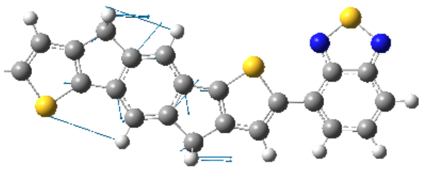

<table>
<tr><th>Cation radical
Expt. $\bar{\nu}$ (Calc. $\bar{\nu}$, chloroform)</th><th>Anion radical
Expt. $\bar{\nu}$ (Calc. $\bar{\nu}$, chloroform)</th></tr>
<tr>
<td>1574 cm^{-1} (1668 cm^{-1})
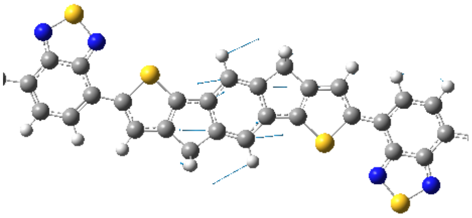
</td>
<td>1596 cm^{-1} (1696 cm^{-1})
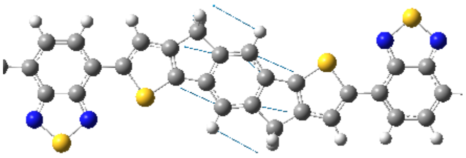
</td>
</tr>
<tr>
<td>1412 cm^{-1} (1500 cm^{-1})
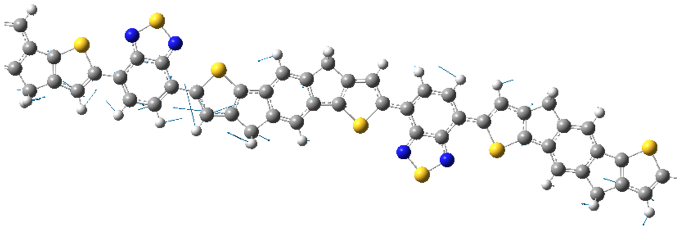
</td>
<td>1523 cm^{-1} (1612 cm^{-1})
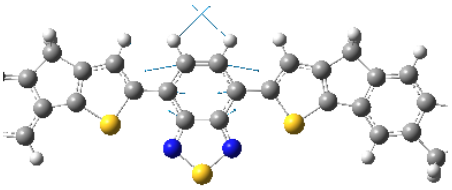
</td>
</tr>
<tr>
<td>1289 cm^{-1} (1361 cm^{-1})
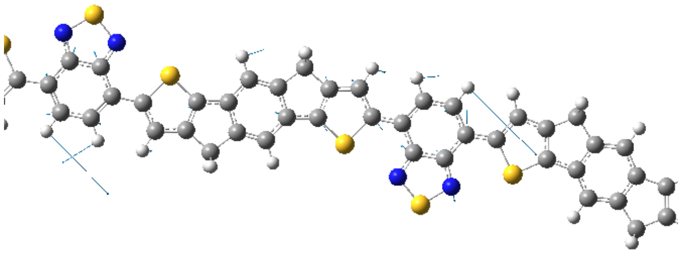
</td>
<td>1412 cm^{-1} (1507 cm^{-1})
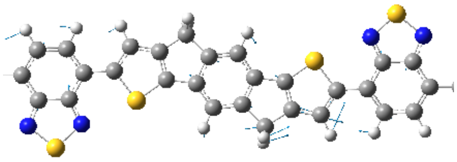
</td>
</tr>
<tr>
<td>1215 cm^{-1} (1256 cm^{-1})
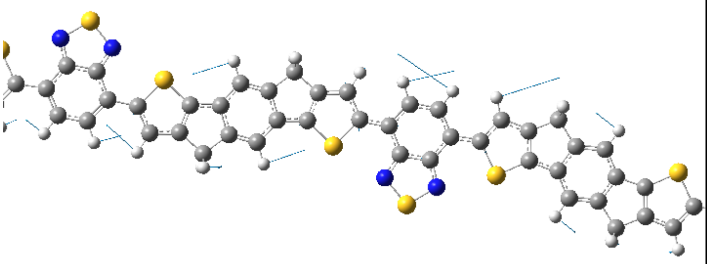
</td>
<td>1373 cm^{-1} (1464 cm^{-1})
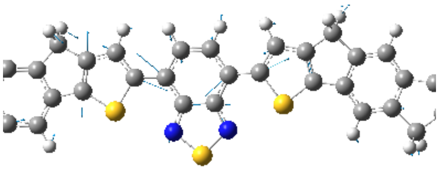
</td>
</tr>
<tr>
<td></td>
<td>1289 cm^{-1} (1365 cm^{-1})
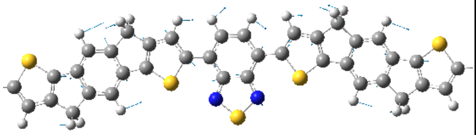
</td>
</tr>
</table>

**S_1 IR**<br>Expt. $\bar{\nu}$ (Calc. $\bar{\nu}$, chloroform)
1581 cm^{-1} (1681 cm^{-1})<br>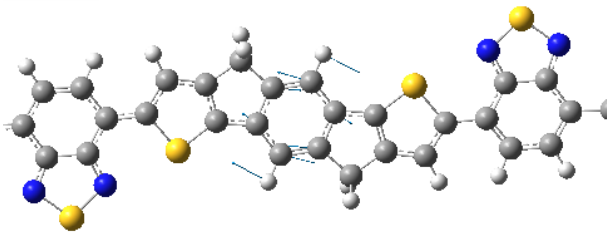
1512 cm^{-1} (1609 cm^{-1})<br>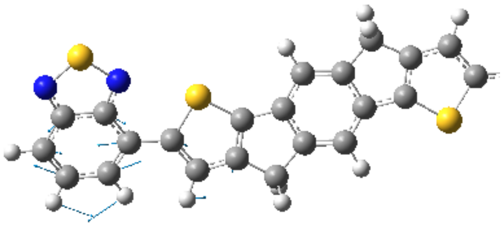
1435 cm^{-1} (1530 cm^{-1})<br>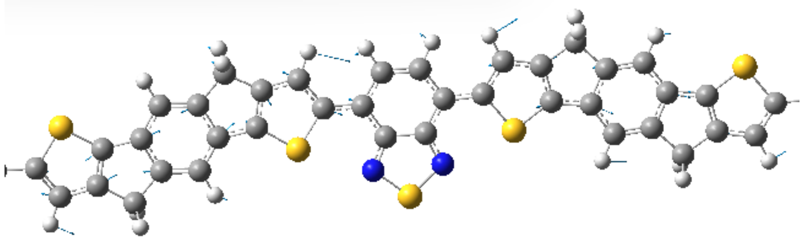
1373 cm^{-1} (1470 cm^{-1})<br>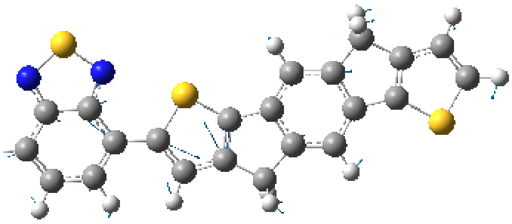
1335 cm^{-1} (1437 cm^{-1})<br>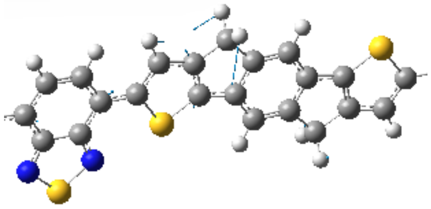

6. Excited state absorption spectra.

The energies and oscillator strength of the 20 first excited state transitions upon absorption from the ground state, cation and anion are calculated using the TDDFT option in Gaussian 16. The TDDFT calculations are done in the optimized geometry for each state of the trimer (ground state, Cation or Anion).

For the excited state absorption calculation, we use the Multiwfn program to calculate the transition dipole moment between the different excited states of the trimer.[2] We use for this the TDDFT output from Gaussian of the trimer in the optimized geometry for the ground state. The absorption spectra in figure S11 are computed using the different transitions presented in table S5. For each state of the molecule, for example the absorption of the first excited state is calculated considering the different electronic transitions shown in table S5 from the S_1 to a higher energy state. The contribution of each electronic transition (from S_1 to a higher electronic state) is calculated as the product of the square of the transition dipole moment (representing the strength of the optical transition) and a Gaussian centred around the energy of the transition to broaden the spectra and consider the low frequency vibrational mode contributions to the absorption spectra. The spectrum for the absorption from the S_1 state is calculated as

$$A_{S1}(\hbar\omega) = A_{S1\rightarrow S2} + A_{S1\rightarrow S3} + \cdots = \sum_i M_i^2 \, exp\left(\frac{(\hbar\omega - E_i)^2}{\sigma^2}\right)$$
$$= \sum_i 32 \frac{\hbar^2}{E_i m_e} f_{osc_i} \;\; exp\left(\frac{(\hbar\omega - E_i)^2}{\sigma^2}\right) \tag{6}$$

where M_i , E_i and f_{osc_i} are the transition dipole moment, Energy and oscillator strength for the electronic transition considered, m_e is the mass of the electron, $\hbar$ is the reduced Planck constant. σ^2 is the broadening factor.

Table S5 Ground state, excited state, cation and anion absorption of a trimer of IDTBR calculated using TDDFT. CAM-B3LYP exchange-correlation functional with the cc-pVDZ basis set. For the excited state absorption, the initial and final state indicate transition from which excited state to the final one.

Ground state absorption			Excited state absorption (i for initial state, and f for final state)				Cation Absorption			Anion absorption		
Excited state	Energy (eV)	Oscillator strength	i	f	Diff.(eV)	Oscillator strength	Excited state	Diff.(eV)	Oscillator strength	Excited state	Diff.(eV)	Oscillator strength
1	2.38	3.60	1	2	0.06	$4\ 10^{-5}$	1	0.57	2.41	1	0.49	0.61
2	2.60	0.00	1	3	0.17	$1\ 10^{-3}$	2	1.10	0.05	2	0.84	0.21
3	3.04	0.42	1	4	0.25	$5\ 10^{-3}$	3	1.29	0.60	3	1.07	0.10
4	3.04	0.02	1	5	0.33	$2\ 10^{-3}$	4	1.42	0.69	4	1.39	0.18
6	3.70	1.50	2	3	0.11	$1\ 10^{-3}$	6	1.71	0.02	6	1.67	0.12
7	3.82	0.01	2	4	0.19	$2\ 10^{-4}$	7	1.97	0.43	7	1.80	0.92
8	3.93	0.02	2	5	0.27	$7\ 10^{-3}$	8	2.11	0.01	8	1.96	0.06

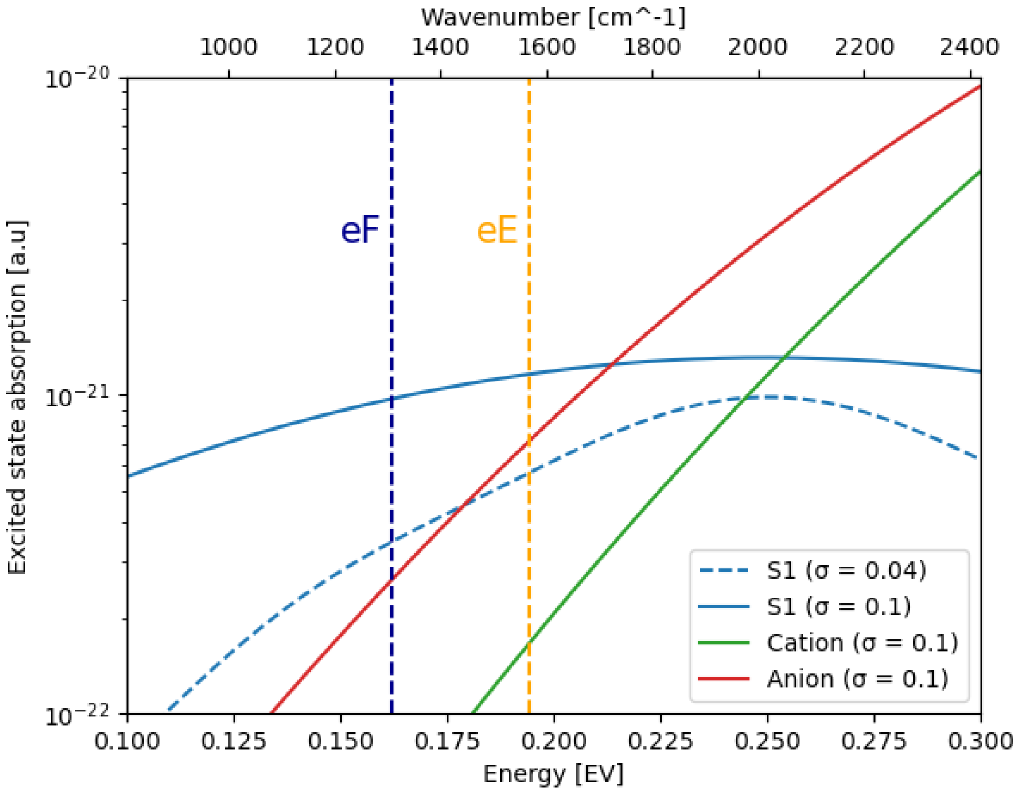

Figure S12: Simulated mid IR absorption spectra of the excited states of a trimer of IDTBR in vacuum. In this case we used the calculation in table S4 and broadened the absorption peaks with a gaussian function with different broadening factors. eE and eF spectral positions are shown as vertical lines.

Although the calculations show a strong contribution of both species to the electronic absorption, the calculations do not agree with the relative contribution of the species at the two positions eE (1568 cm^{-1}) and eF (1308 cm^{-1}). Depending on the broadening factor considered for the different transitions, we can have different relative contributions of the states to the electronic band positions identified in the main text. This discrepancy between calculated and experimental relative intensities can be attributed to several factors inherent to computational modelling of electronic absorption in conjugated polymers. First, the choice of exchange-correlation functional and basis set significantly affects the accuracy of predicted transition energies and oscillator strengths. Second, the truncation of the polymer chain to a trimer model may not fully capture the extended electronic delocalization present in longer chains, potentially altering the relative intensities of electronic transitions. Third, solvent effects and conformational disorder in solution, which are approximated in the calculations, can substantially influence the electronic absorption spectrum. Finally, the experimental broadening of electronic transitions in solution differs from the Gaussian broadening applied in the theoretical spectra, leading to variations in the apparent relative intensities at specific wavenumber positions.

7. Model for charge generation

In this section we present a 3-state kinetic model to explain the experimental observation of the paper and their implication for the charge generation process in the studied polymer. The model considers that the photon is absorbed into a manifold of excitons (S_1). As we are excited close to the band gap, this is the only state absorbing the light. We consider that the polaron does not absorb light. So, the initial population of the states is $n_{S_1} = 1$, $n_p = 0$ and $n_{GS} = -1$. Here n_{S_1}, n_p and n_{GS} are the population of the S_1, polaron and ground state respectively. The S_1 state can then either decay to the ground state or form a polaron, following

$$\frac{dn_{S1}}{dt} = \frac{n_p}{\tau_{p,S1,}} - \frac{n_{S1}}{\tau_{S1,p}} - \frac{n_{S1}}{\tau_{S1,GS}} \qquad (7)$$

where τ_{p,S_1} is the time constant for the exciton reformation from the polaron, $\tau_{S_1,p}$ is the exciton dissociation time constant, and $\tau_{S_1,GS}$ is the decay of the S_1 to the ground state time constant. In this model we consider that the polaron can either reform the S_1 state or decay to the ground state following

$$\frac{dn_p}{dt} = -\frac{n_P}{\tau_{p,S_1}} + \frac{n_{S1}}{\tau_{S_1,p}} - \frac{n_P}{\tau_{\mathrm{p,GS}}} \qquad (8)$$

where $\tau_{\mathrm{p,GS}}$ is the time constant for the recombination of the polaron to the ground state. Therefore, the change in the ground state population is related to the rate of S_1 and polaron decay to ground

$$\frac{dn_{GS}}{dt} = \frac{n_{S_1}}{\tau_{S_1,GS}} + \frac{n_P}{\tau_{\mathrm{p,GS}}}. \qquad (9)$$

To reproduce the experimental traces, we consider that the temporal evolution of the IR peaks can be reproduced as:

$$\Delta OD_{vib,\bar{\nu}}(t) = \sigma_{\bar{\nu},p}\, b\, n_p(t) + \sigma_{\bar{\nu},S_1}\, b\, n_{S_1}(t) - \sigma_{\bar{\nu},GS}\, b\, n_{GS}(t) \qquad (10)$$

where $\sigma_{\bar{\nu},p}, \sigma_{\bar{\nu},S_1} and\ \sigma_{\bar{\nu},GS}$ are the absorption cross sections at wavenumber $\bar{\nu}$ of the polaron, S_1 and ground state, respectively, and *b* is the cell pathlength (250 µm). For the baseline absorption related to the electronic absorption in the IR region of the different excited states, we consider

$$\Delta OD_{baseline,\bar{\nu}}(t) = \sigma^{*}_{\bar{\nu}.S_1}\, b\, n_{S_1}(t) + \sigma^{*}_{\bar{\nu},p}\, b\, n_p(t) \quad (11)$$

with the contribution of the different excited states to the baseline spectra adapted considering the lifetimes of the different transitions. Here $\sigma^{*}_{\bar{\nu}.S_1}, \sigma^{*}_{\bar{\nu},p}$ are the absorption cross sections at wavenumber $\bar{\nu}$ of the baseline for the S_1 and polaron states, respectively.

7.1. Reproducing the experimental data

Considering the model presented above, the number of free parameters to reproduce the experimental data rises to 29: 4 different time constants, 21 (7 vibrational peaks * 3 absorption cross sections), and 4 baseline absorption cross sections.

To reproduce the experimental data, we first reduce the number of free parameters and introduce constraints to others:

1. For the vibrational peaks, we consider that any peak not showing a negative feature has no contribution from the ground state. This concerns the vibrational peaks vA2, vA3, vA4 and vA5 (associated with wavenumbers 1334, 1375, 1525, 1575 cm^{-1}), reducing the number of free parameters by 4.

2. For the contribution of the excited states (S1 and polaron) to the bleach we consider that the polaron does no contribute to vB1 and vB2 (1260 and 1360 cm^{-1}), and the S_1 does not contribute to vB3 (1394 cm^{-1}), reducing the number of free parameters by 3.

Following these constraints the number of free parameters is 21 (3 time constants and 18 absorption cross sections).

To reproduce the experimental results, we use a sequential fitting procedure, where we first set the time constants to a certain value, then we fit the rest of the absorption cross sections to reproduce the experimental results. We assess the goodness of the fit using χ^2 defined as

$$\chi^2 = \Sigma \left[\left(O_{\bar{\nu},t} - E_{\bar{\nu},t} \right)^2 \right] \tag{12}$$

where: $O_{\bar{\nu},t}$ is the observed kinetic data at wavenumber $\bar{\nu}$ and time *t,* and $E_{\bar{\nu},t}$ is the simulated (model-predicted) value at wavenumber $\bar{\nu}$ and time *t.*

We do a grid exploration of the parameter space of the 4 different time constants within the ranges: 0.1 to 10 ps for $\tau_{S_1,P}$and τ_{p,S_1} and 100 to 10000 ps for $\tau_{S_1,GS}$ and $\tau_{p,GS}$. In figure S12 we show the distribution of the parameters for the samples within 10% of the lowest χ^2 achieved (we found 0.63) and compare it to the distribution of parameters explored overall (100,000 different sets). We find here that in order to achieve a good reproduction of the observations, we need to consider: 1) $\tau_{S_1,P}$ is in the order of 4 ps, which correspond to the rise of the different kinetic traces discussed in the main text; 2) $\tau_{p,S_1}$is in the order of 20-100 ps. 3) The time constants related to the recombination to ground are in the order of 1 ns. The distribution of $\tau_{S1,GS}$ is broader, than that of $\tau_{p,GS}$; meaning that with the current observation we have a larger error in determining $\tau_{S_1,GS}$. The mean and standard deviation of the time constant values that give a good fit to the data are presented in table S6.

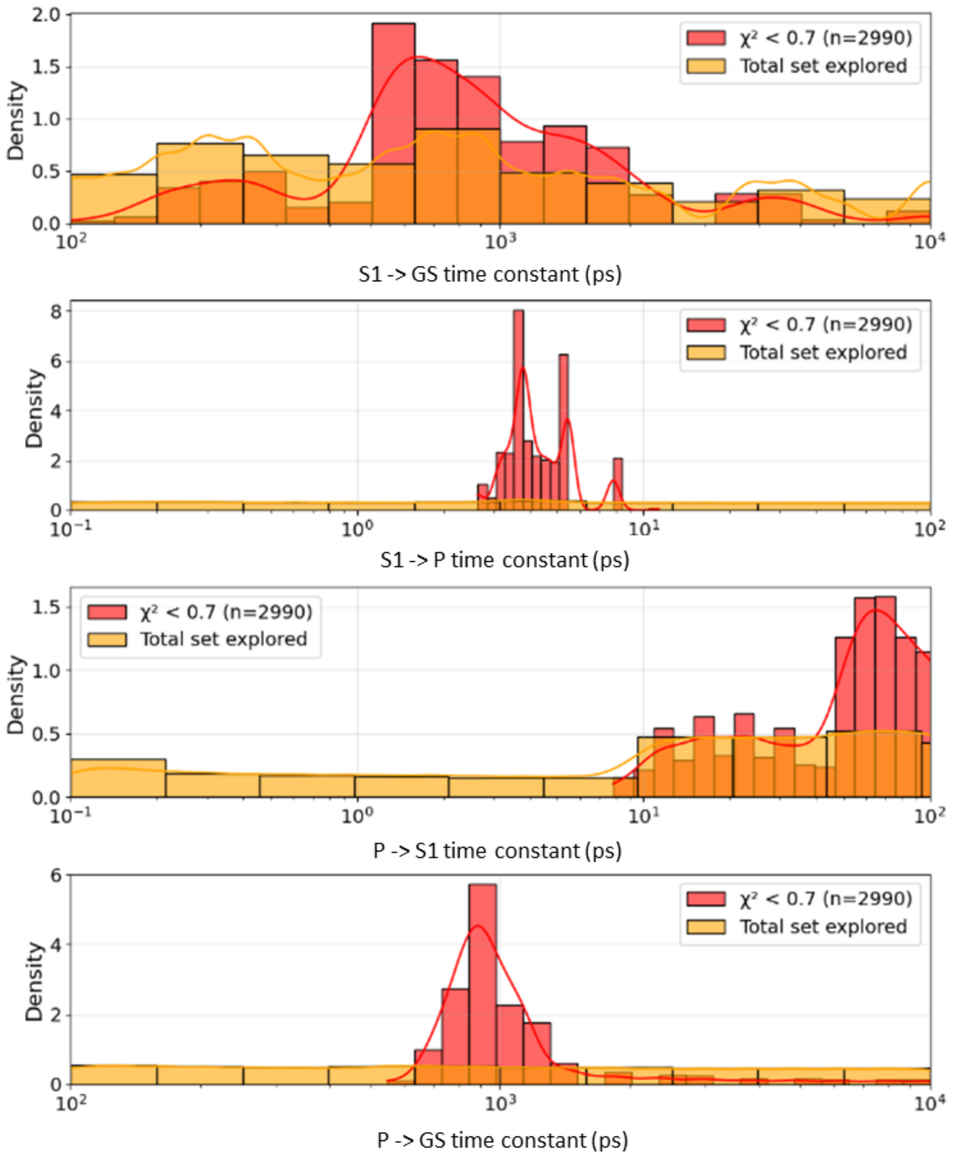

Figure S 13 Parameter exploration to fit the observations in C8-IDTBT in solution. Here we show the histogram of the four different time constants, for two cases: 1) χ<0.7 showing the distribution of parameter sets the accurately reproduce the observations.2) the total set of parameters explored.

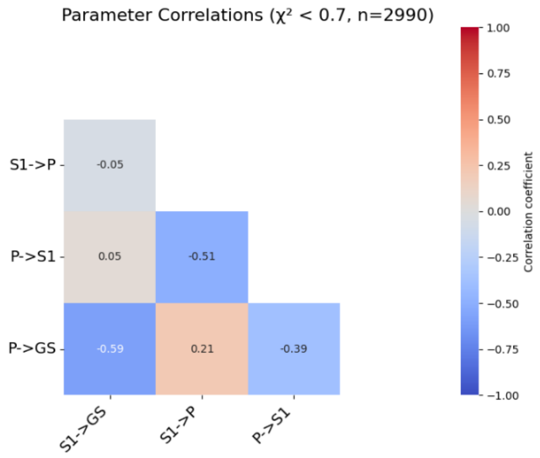

Figure S 14 Correlation plot between the time constants for the sets that fit the observations for C8-IDTBT in solution. We consider here the Pearson correlation where a value close to 1 means a strong positive correlation and a value close to -1 means a strong anti-correlation.

Table S 6 Time constants for C8-IDTBT in solution inferred from reproducing the experimental results. Here the standard deviation is calculated from the distribution in figure S12.

Parameter	Unit	Mean Value	Standard deviation	Ranges
$\tau_{p \to GS}$	ps	1270	1301	0.1 to 5 ns
$\tau_{S_1 \to GS}$	Ps	1100	1400	0.6 to 4 ns
$\tau_{S_1 \to p}$	ps	4.42	1.25	3 to 6 ps
$\tau_{p \to S_1}$	ps	72	47	10 to 100 ps

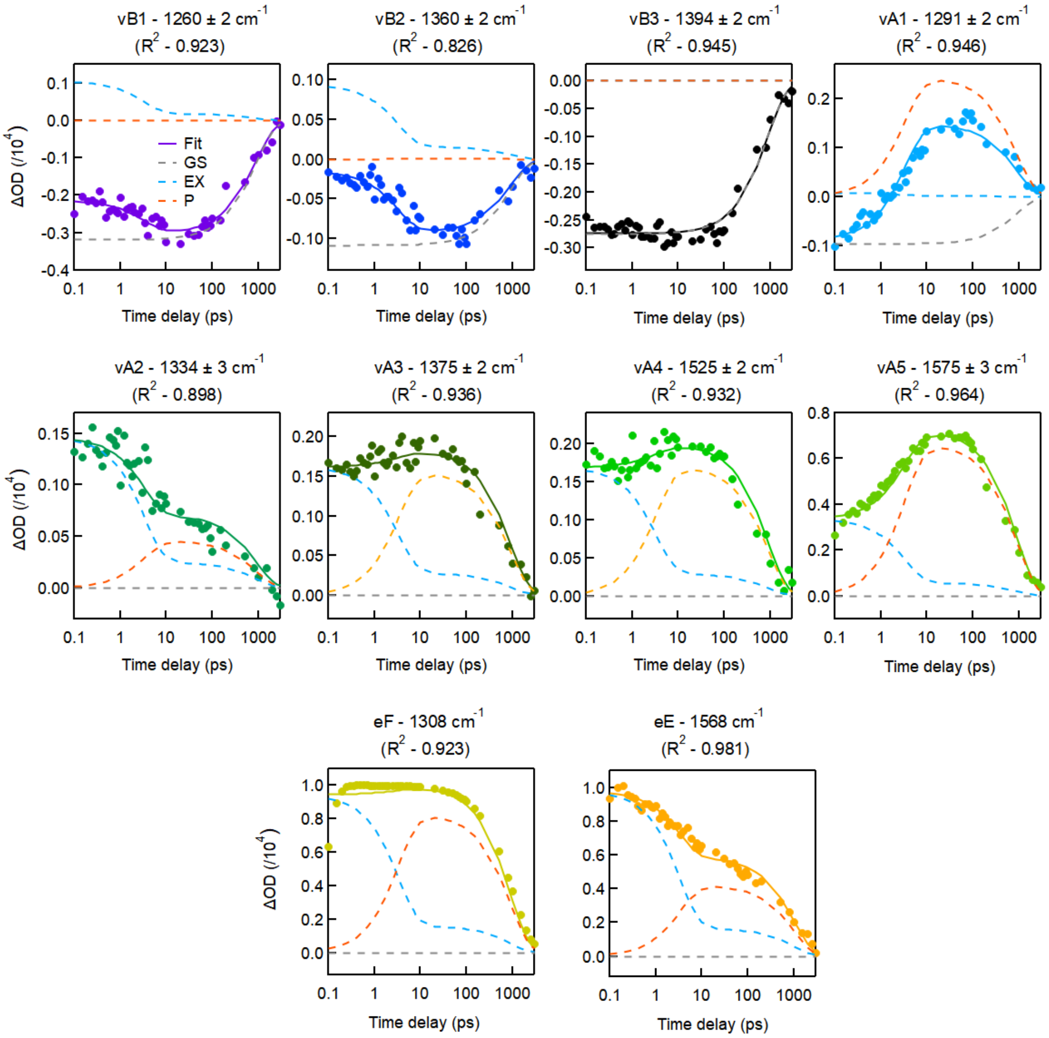

Figure 15 Fit of the experimental kinetic data for the various peaks identified in the study, including vibrational features (vA1–vA5 and vB1–vB3) and broad electronic absorption peaks (eE and eF). The contributions of different electronic states to each kinetic component are indicated with dotted lines: blue represents the excited singlet state (S_1), orange corresponds to the polaronic state, and grey denotes the ground state. The model results shown in this figure are with the following time constants: $\tau_{S_1,GS}$ = 3 ns, $\tau_{S_1,P}$= 4 ps and τ_{P,S_1}=20 ps.

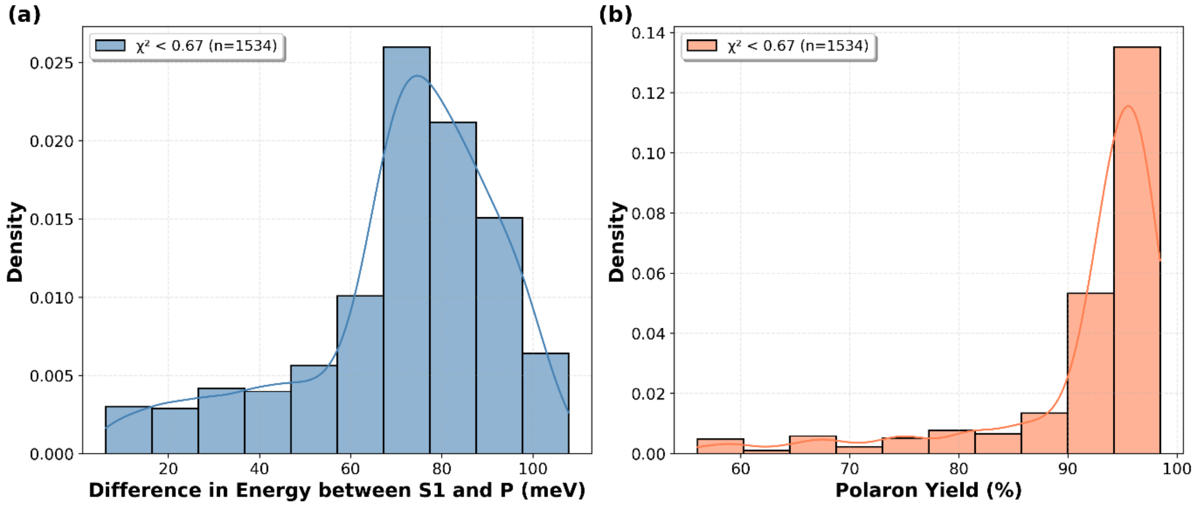

Figure S16 Distribution of (a) the energy difference between exciton and polaron states and (b) the polaron generation yield inferred from the distribution of time constants that reproduce the experimental observations as shown in figure S13. In b), the polaron yield is estimated as the maximum ratio of polarons generated during the transient response of the solution to the photo-excitation.

7.2. Charge extraction efficiency

In this section we explore the relationship between polaron generation and the charge generation efficiency that could result from the extraction of polaron pairs to an external circuit. To do this, we add to the model an extraction pathway from the polaron pair state to an external circuit with an extraction rate constant of τ_{ext}. In steady state conditions, we estimate the charge generation efficiency as the ratio of the flux of extracted charge carriers, n_p/τ_{ext}, to the initial photogeneration rate.

Figure 16 shows the charge generation efficiency as a function of τ_{ext}, with all other model parameters as set in Table S6. To achieve a yield of charge generation efficiency above 10 %, the extraction should occur in less than 10 ns. We can relate τ_{ext} to the charge carrier mobility μ as follows. First we consider the polaron size a_p to be around 10 nm (6 repeat units of the polymer), and that the charge needs to move for at least its length, here 10 nm, to become free. Under normal solar cell operation and a film thickness of 100 nm, the applied field Φ is on the order of 10^4 Vcm^{-1} at operating point, reaching a maximum of around 10^5 Vcm^{-1} at short circuit. The hole has a mobility μ, whose value for C8-IDTBT will be in the range of 0.1 (approximate value of its reported FET hole mobility in Ref 3 to 1 cm^2 /Vs (intrachain mobility of the better performing polymer C16-IDTBT estimated at up to 3 cm^2 /Vs from THz

measurements [4]. We now consider the time τ_{ext} for a hole to escape from the polaron pair in two limits, (1) in the absence and (2) in the presence of Coulomb interactions between the electron and hole polaron on the chain.

In case (1) the charge extraction time constant can be calculated as: $\tau_{ext} = \frac{a_p}{\Phi * \mu}$, where μ is the charge carrier mobility. Taking an intermediate value of the $\Phi\mu$ product as 10^4 cm s^{-1} the escape time becomes around 100 ps, which according to Figure S16 would allow significant charge generation. However, this treatment neglects the impact of Coulomb interaction on the charge escape. In case (2), we estimate the Coulomb attraction energy between the charges to be at least 0.1 eV assuming a relative permittivity of $\epsilon_r = 4$ and an electron-hole separation of a_p (10 nm) at the maximum. In this condition, a field of several times larger than that experienced in a solar cell would be needed before τ_{ext} would enter the regime where charge generation is significant. This argument is compatible with experimental measurements on C16-IDTBT that show that photocurrent is not collected from polymer photodiodes at fields less than 10^6 V cm^{-1}.

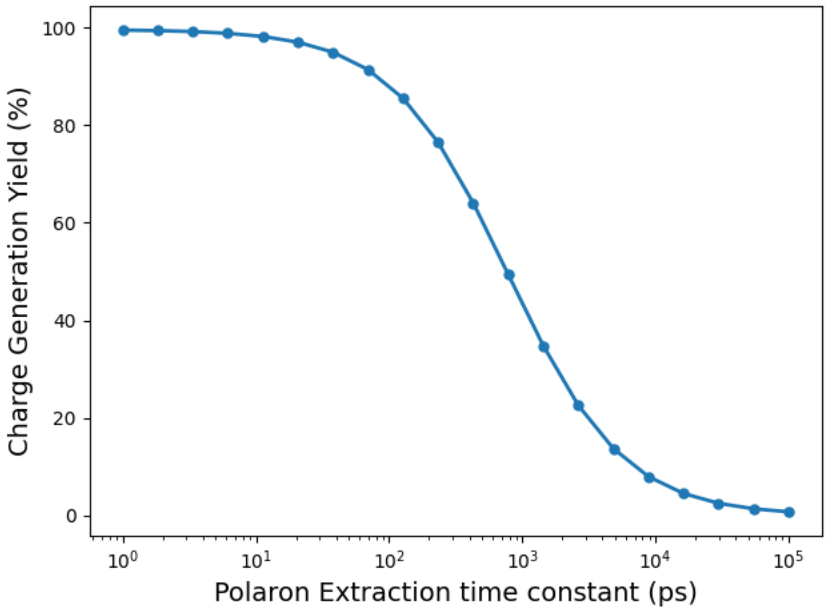

Figure S17 Charge generation yield as a function of the polaron extraction time constant using the values of the time constant in table S6.

7.3. TRIR data for C8-IDTBT film.

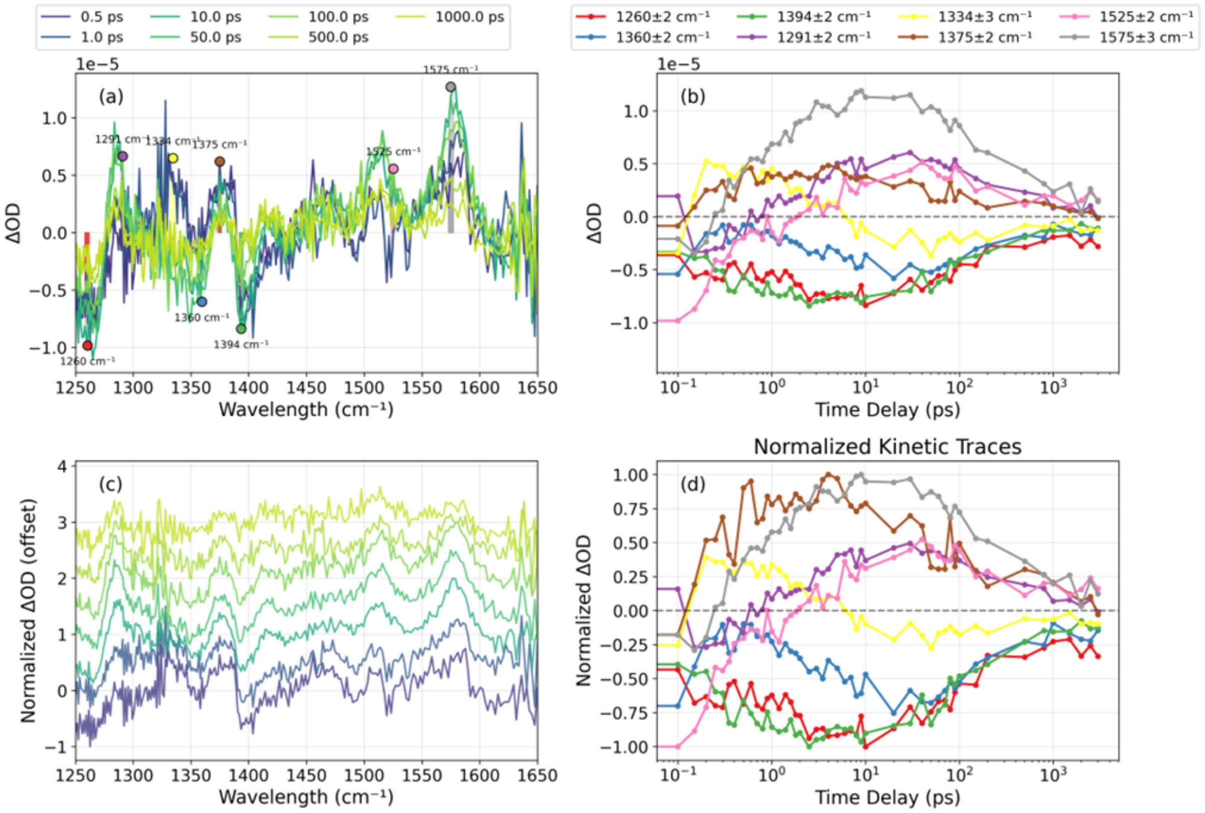

Figure S18 HMW C8-IDT-BT film transient IR spectroscopy data overview. (a) Transient IR spectra at representative time delays with marked wavenumber regions for kinetic analysis (colored circles). (b) Raw kinetic traces at selected wavenumbers. (c) Normalized spectral evolution with vertical offset. (d) Normalized kinetic traces on a logarithmic time scale. Time delays are in picoseconds, wavenumbers in cm^{-1}, and signal intensity as change in optical density (ΔOD).

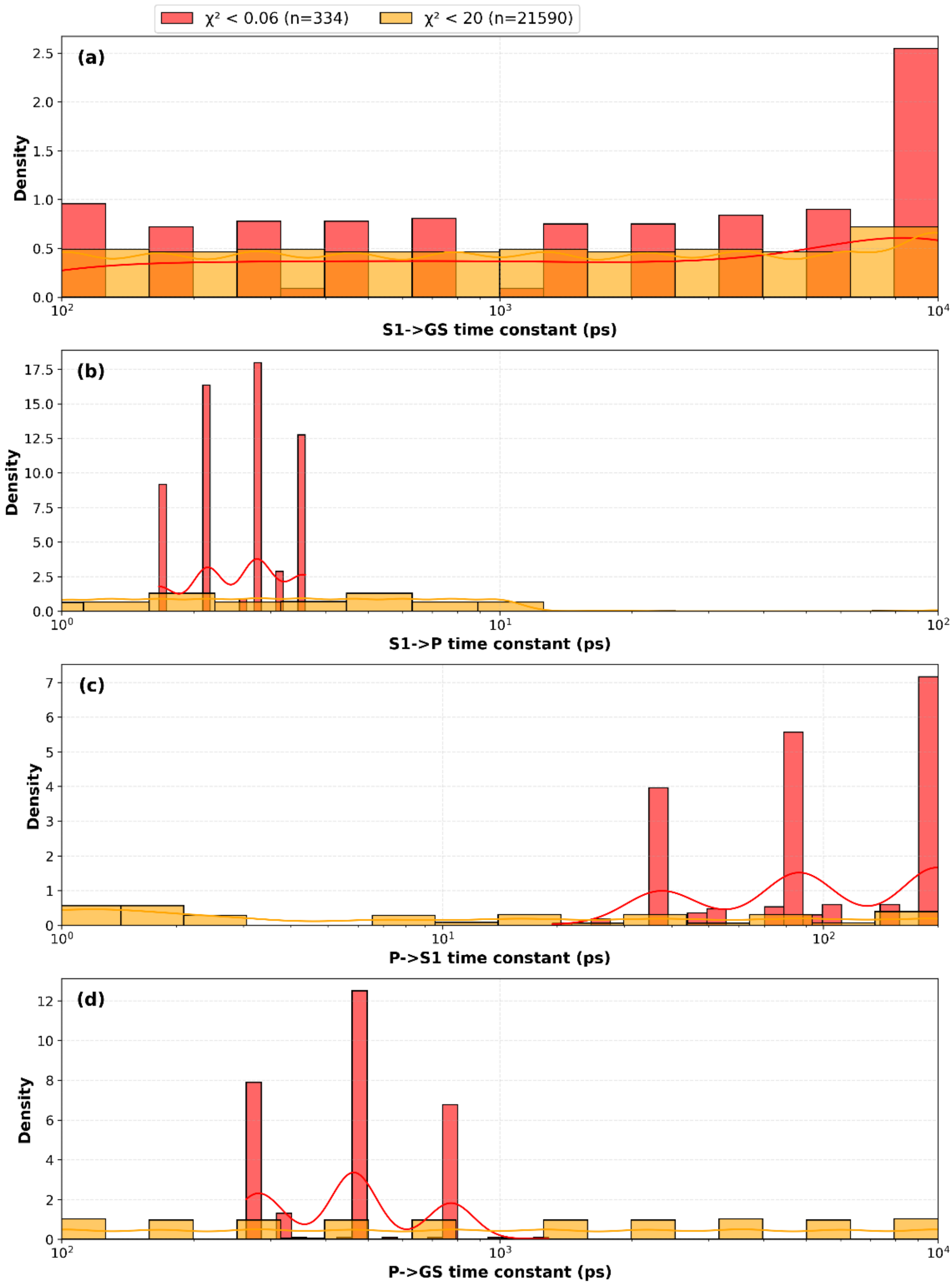

Figure S19 Parameter exploration to fit the TRIR measurement of a HMW C8-IDTBT film. Here we show the histogram of the four different time constants, for two cases: 1) χ<0.06 showing the distribution of parameter sets the accurately reproduce the observations. 2) the total set of parameters explored.

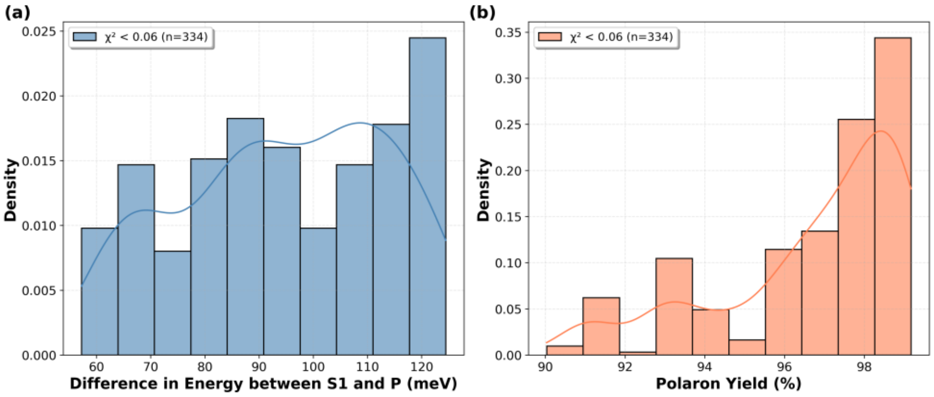

Figure S20 Distribution of (a) the energy difference between exciton and polaron states and (b) the polaron generation yield inferred from the distribution of rate constants that reproduce the experimental observations as shown in figure S18. In b), the polaron yield is estimated as the maximum ratio of polarons generated during the transient response of the solution to the photo-excitation.

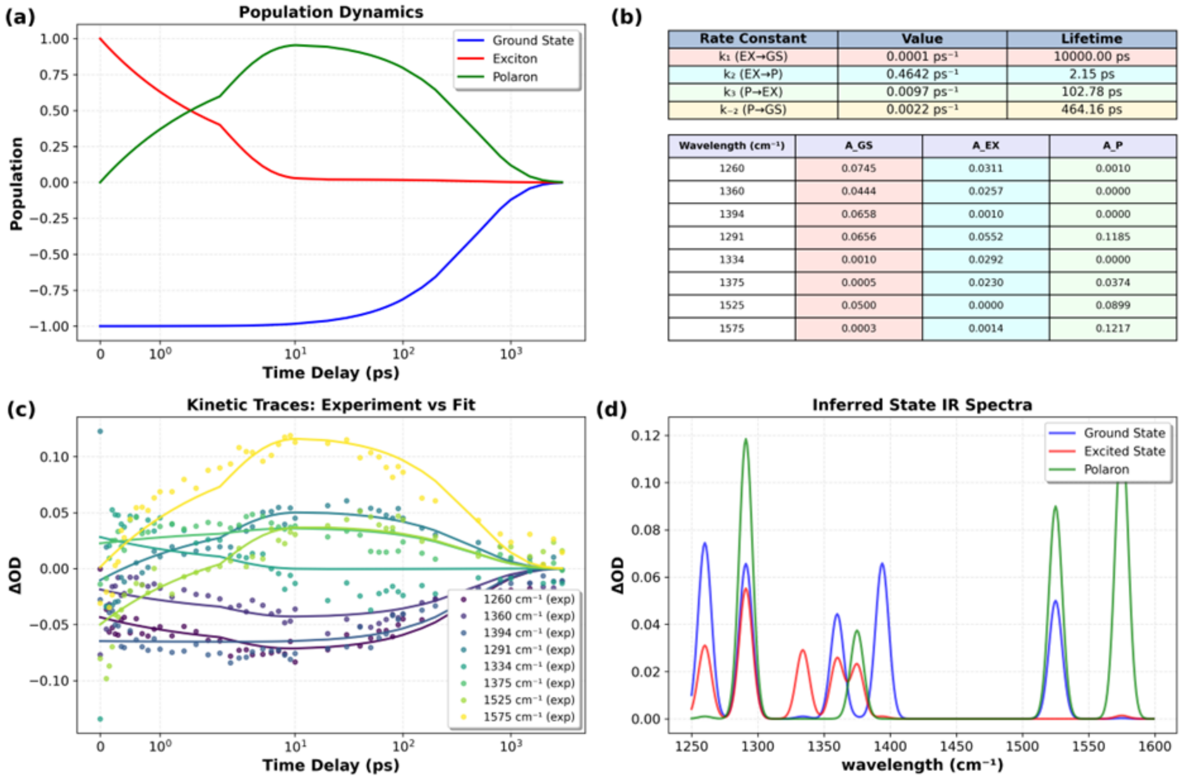

Rate Constant	Value	Lifetime
k_1 (EX→GS)	0.0001 ps^{-1}	10000.00 ps
k_2 (EX→P)	0.4642 ps^{-1}	2.15 ps
k_3 (P→EX)	0.0097 ps^{-1}	102.78 ps
k_{-2} (P→GS)	0.0022 ps^{-1}	464.16 ps

Wavelength (cm^{-1})	A_GS	A_EX	A_P
1260	0.0745	0.0311	0.0010
1360	0.0444	0.0257	0.0000
1394	0.0658	0.0010	0.0000
1291	0.0656	0.0552	0.1185
1334	0.0010	0.0292	0.0000
1375	0.0005	0.0230	0.0374
1525	0.0500	0.0000	0.0899
1575	0.0003	0.0014	0.1217

Figure S21 Three-state kinetic model fitting results for transient IR spectroscopy results for a HMW C8-IDTBT film. (a) Population dynamics of the three states (Ground State, Exciton, and Polaron) as a function of time delay. (b) Top: Fitted rate constants with corresponding lifetimes for all transitions (k_1: EX→GS, k_2: EX→P, k_3: P→EX, k_{-2}: P→GS). Bottom: Selected spectral amplitudes (A_GS, A_EX, A_P) at representative wavenumbers. (c) Comparison of experimental kinetic traces (markers) and fitted model (solid lines) at selected wavenumbers, demonstrating the quality of the fit across the spectral range. (d) Inferred IR absorption spectra for each state, reconstructed from the fitted spectral amplitudes using Gaussian smoothing (σ = 5 cm^{-1}).

8. Calculation of the polaron quantum yield

The quantum yield (QY) of polarons generated in our TRIR experiment can be calculated when the maximum intensity of a characteristic cation band is compared to the intensity of a ground state bleach band in the same time delay spectrum, as these intensities are proportional to the concentrations of the generated and lost species following

$$QY = \frac{[cation\ radical]}{[ground\ state]} = \frac{A_{cation}/(\varepsilon_{1574} \times b)}{A_{GSB}/(\varepsilon_{1394} \times b)} \quad (13)$$

This requires knowledge of the molar extinction coefficients (ε) for the two characteristic bands. The molar extinction coefficient for the main 1574 cm^{-1} cation radical band (vA), assigned to the C=C stretch of the IDT unit, needs to be determined from an accurate measurement of the charge density in a sample. We choose to use the CMS spectra, where the charge density is controlled entirely by the dielectric capacitance and the applied voltage. In this measurement, polarons occupy the interface of a dielectric, forming a thin accumulation layer of ~1 nm thickness as seen in the following schematic (Figure S21). [5, 6]

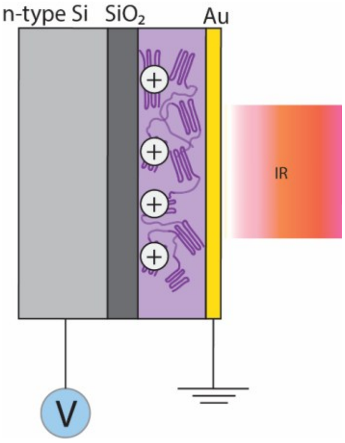

Figure S22: Schematic of the CMS sample measurement setup.

The absorption cross section, $\sigma(\bar{v})$, can be calculated from the differential transmission (ΔT/T) (reported as the change in transmission of a sample going from higher charge density (T_+) to a lower charge density (T_-), normalized by the neutral sample transmission (T_0)), according to (Figure S22):

$$\frac{\Delta T(\bar{v})}{T_0(\bar{v})} = \frac{T_+(\bar{v}) - T_-(\bar{v})}{T_0(\bar{v})} = -C_{areal} e^{-1} \Delta V \sigma(\bar{v}) \approx -\Delta\alpha(\bar{v}) D \quad (14)$$

where e is the electron charge in C, ΔV is the applied voltage, C_{areal} is the areal capacitance (here 2.3 x 10^{-8} F cm^{-2}), D is the accumulation layer thickness and $\Delta\alpha(\bar{v})$ the differential absorption coefficient. With an applied voltage of 40 V and an accumulation layer of 1 nm, a volumetric charge density (N/V) of ~5.75 x 10^{19} cm^{-3} was obtained.

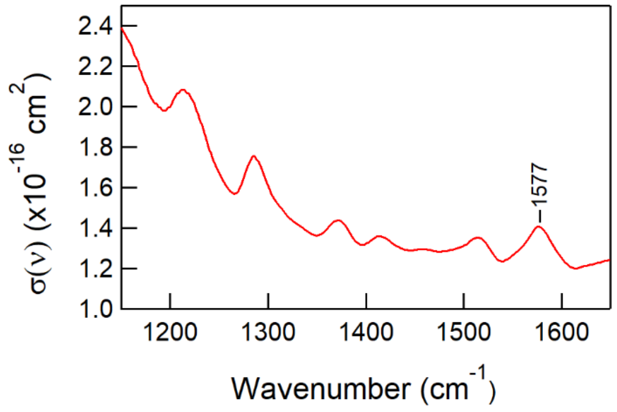

Figure S23: Absorption cross section of the low molecular weight C_{16}-IDTBT film measured through charge modulation spectroscopy.

From the CMS spectrum in Figure S10, we can obtain the absorption cross section for the 1577 cm^{-1} band (found equal to 0.2 × 10^{-16} cm^2/molecule from the height of the peak) and then convert to molar extinction coefficient through:

$$\varepsilon\ (cm^{-1}M^{-1}) = \frac{\sigma(\bar{v})N_A}{2.303} \times 10^{-19} \qquad (15)$$

where the cross section is in units of $Å^2$/molecule. This gives an ε = 5230 $cm^{-1}M^{-1}$. A similar value is obtained (ε = 5199 $cm^{-1}M^{-1}$) if one uses instead the volumetric charge density, $N/_V$, (found to be equal to 5.75 x 10^{19} cm^{-3}) in the following equation:

$$\varepsilon(cm^{-1}M^{-1}) = \frac{\Delta\alpha(\bar{v})}{2.303} \times \frac{MW}{\rho} \times 10^{-3} = \frac{\Delta\alpha(\bar{v})}{2.303} \times \frac{N_A}{N/_V} \times 10^{-3} \qquad (16)$$

Using the latter molar extinction coefficient, a cation radical concentration of 4.5×10^{-7} M was calculated from the 10 ps TRIR spectrum of C8-IDTBT.

The extinction coefficient for the 1390 cm^{-1} band from the GS IR spectrum, was determined ratiometrically with respect to the visible absorption spectrum of the same film (Eq. 16, Figure S23), as the molar extinction coefficient for the latter band was known (ε_{666nm} = 93335 $cm^{-1}M^{-1}$). This way we avoided having to know the film thickness (b) or concentration (C) of the

polymer in the film. This procedure gave a molar extinction coefficient $\varepsilon_{1390\ cm^{-1}}$ = 1587 cm^{-1}M^{-1}. Then using Eq. 13 above gave a QY for polaron generation equal to 64%.

$$\frac{A_{1390\ cm^{-1}}}{A_{666\ nm}} = \frac{\varepsilon_{1390\ cm^{-1}} \times b \times C}{\varepsilon_{666\ nm} \times b \times C} \tag{17}$$

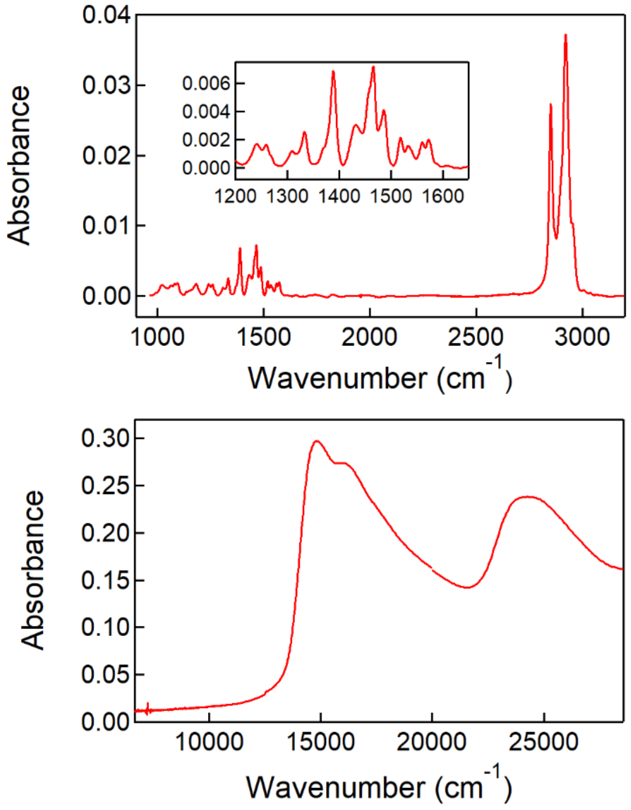

Figure S24: (Top) Ground state FTIR and (bottom) UV-vis absorption spectra obtained for the same thin film of C16-IDTBT.

The error for the calculation of the QY for polaron formation can be estimated based on the error for the measured differential extinction coefficient from the CMS experiment. Above, the charge density was considered constant within 1 nm of the semiconductor layer. However, if we assume a distribution of charges in the accumulation layer then a range of thicknesses needs to be considered. According to Tanase et al, ~92% of the charge density is expected within the first 1 nm of the semiconductor layer.[7] Thus, if we estimate the differential extinction coefficient considering a range of thicknesses from 0.75 – 1.3 nm, the QY is found to range from 48 – 83%. In addition, comparing the CMS with the TRIR spectra (Figure S6) we can see that the CMS spectra are of lower resolution. The polaron extinction

coefficient was estimated based on the height of the CMS peak, which due to the lower resolution could underestimate this extinction coefficient. If we consider the FWHM for the 1576 cm^{-1} peak (31 cm^{-1}) in the CMS spectrum vs. the TRIR (18 cm^{-1}), we could estimate an extinction coefficient of ~9000 $cm^{-1}M^{-1}$ and a QY of 37%. Furthermore, as also mentioned in the main text, an estimation of the QY can also be made based on the extent of the ground state bleach in the TRIR spectra right after excitation and at 10 ps delay time, yielding a QY of ~80%. Therefore, based on all the above uncertainties, we can infer a QY for polaron formation of 60 $\pm$20%.

9. Nature of the excited state: looking for the low energy CT state.

Here we investigate the presence of a low energy excited state that could drive the fast generation of the polaron pair. We use DFT and TDDFT calculations to find a low energy excited state with a strong charge transfer character.[8] We use TheoDore toolbox to analyse the properties of the excited states and assess their charge transfer nature.[9]

In here we consider both the case of intramolecular charge transfer states and intermolecular charge transfer states.

9.1. Intramolecular Excited States.

9.1.1. Ground state geometry:

First, we focus on the presence of a low energy intramolecular charge transfer state. We calculate the excited state of the optimized geometry of the IDTBT 6-mer using cam-b3lyp/cc-pvdz (Table S7). Among the first 10 low energy excited states of the 6-mer, only the ones with energy above 3 eV show a significantly higher electron hole distance (higher than 10 Å), which is related to a stronger charge transfer character.

Moreover, the natural transition orbitals for the first 2 excited states (Figure S24), show a delocalization of the hole and electron wavefunction over similar units. This confirms that these states have a strong excitonic character. Here the first two excited states in the 6-mer are part of the first absorption peak observed, Figure 1 in the main paper. The NTO's related to the second absorption bump are not shown below, as we are mainly interested in the properties of the lowest energy excited states.

Table S7: Calculated transitions from the ground state to various electronic excited states of an IDTBT 6-mer in its optimized geometry. The calculations are done using cam-b3lyp/cc-pvdz

6IDTBR GS absorption			
Excited state	Excited state energy (eV)	Oscillator strength	Average electron hole distance (Å)
1	2.21	8.1	6.895
2	2.31	0.0237	6.748
3	2.43	0.4888	6.531
4	2.53	0.0005	6.328
5	2.61	0.0398	6.19
6	2.99	0.4189	6.488
7	3.24	0.0056	8.751
8	3.26	0.0051	9.515
9	3.30	0.0134	10.885

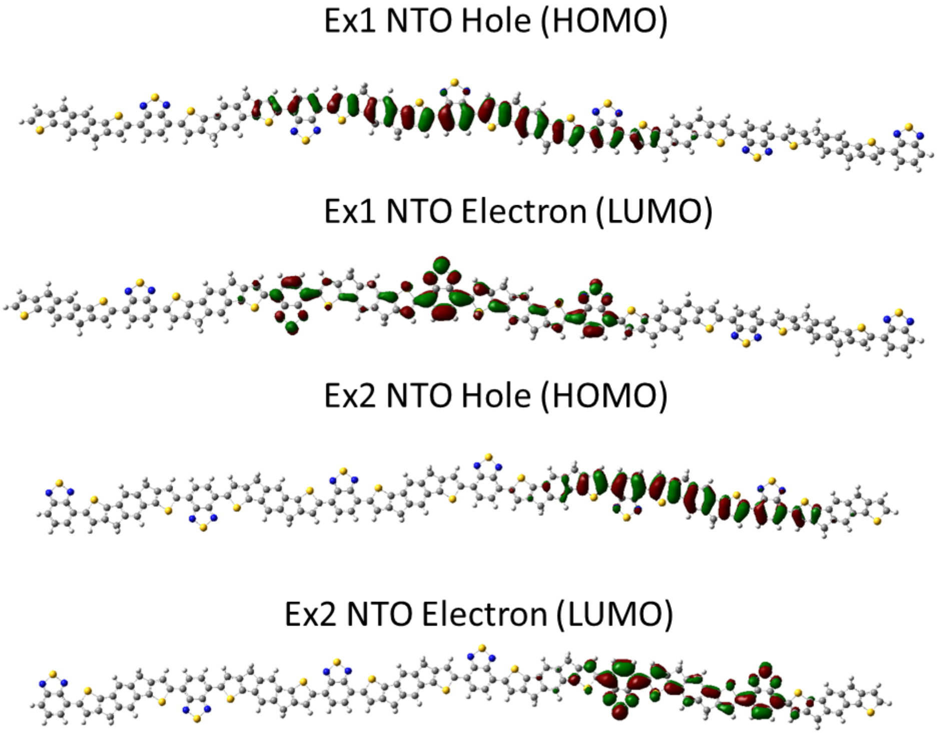

Figure S25: Natural transition orbitals of the first 2 excited states of an IDTBT 6-mer.

9.1.2. MD generated geometries.

We then investigate the potential presence of a low-lying excited state with strong charge transfer character in different IDTBT conformers. Considering the polymer will not necessarily be in their ground state optimised geometry, we used molecular dynamics (MD) to generate an ensemble of different IDTBT chain structures that could be present in solution. To ensure that the different conformers were suitably distinct (i.e., uncorrelated), preliminary chain geometries were first extracted from a 1.5 μs long simulation of a C8-IDTBT 12mer chain immersed in chlorobenzene solvent at 363K. This simulation was performed using a coarse-grained forcefield derived from the MARTINI package.[10] Snapshots of the chain geometry were extracted every 15 ns, producing 100 conformers. These were back mapped to convert from a coarse-grained representation to an atomistic representation based on the OPLS-AA forcefield.[11, 12] Details of both IDTBT forcefields may be found in reference [13]. Each of the now atomistic conformers was then subject to a further MD simulation while immersed in chloroform, which continued for 100 ps at 300 K. To prepare the conformers for input into excited state calculations, they were then shortened to 6mers, by removing 3 monomers from each end, and replacing them with terminal hydrogens. The sidechains were also shortened to methyl groups.

Since there may be small differences between the atomic positions predicted by MD and DFT, and to fix any unnatural bond lengths introduced by editing the structures, each of the conformers was partially optimized (for 3 steps) using DFT at the cam-b3lyp/cc-pvdz level of theory. After this, the conformers were input into TDDFT calculations and the charge transfer character of the excited states was analyzed using TheoDore, following the same procedure applied to the optimized structures. Similar to the results of the optimised geometry, the only excited states showing significant charge transfer character occur at energies higher than 3 eV, which lies 0.6 eV higher than the lowest excitation (Figure S25).

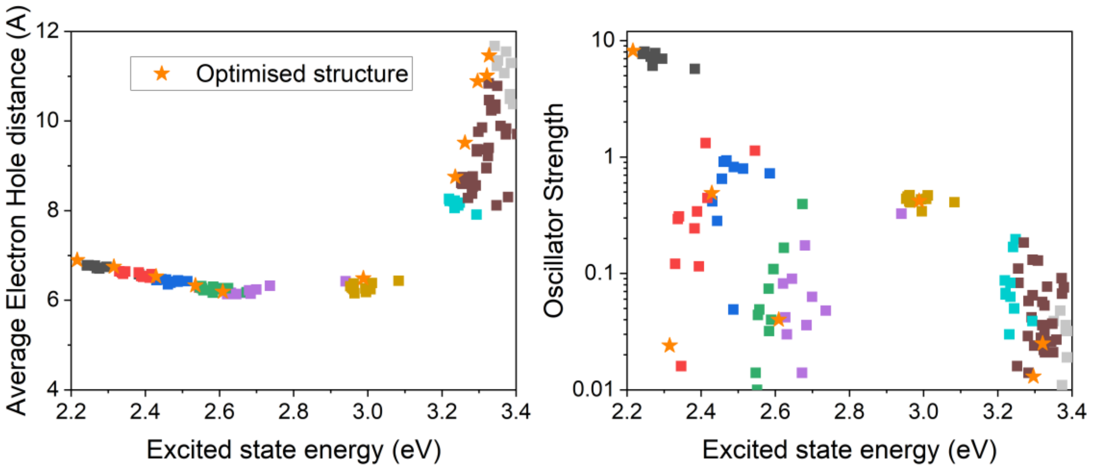

Figure S26: Excited state properties of the first excited states of the MD generated IDTBT 6-mer structures. (a) Average electron hole distance, and (b) oscillator strength as a function of excited state energy. Here each color shows a different excited state manifold, with the star symbol showing the excited states of the molecule in its optimized geometry. For example, the black colored markers correspond to the first excited state energies in different geometries generated in the MD run.

9.1.3. Impact of excited state relaxation

The next thing we investigated is the nature of the excited states following the relaxation in the different electronic states considered, i.e. the ground state and the first excited state. In this case, we compare the electron hole separation for the different excited states in either the ground state optimised geometry (GS, Geom) or the relaxed optimised geometry in the first excited state (S1, Geom) (figure S26). First point to notice is that the energy of the first excited state drops to 1.82 eV, which is considerably different from the energies in the MD generated structure. This means that upon relaxation the molecular geometry changes more than the geometries accessed by the MD runs. The electron and hole separation in the different excited states does not change for the trimer of IDTBT, whether it is in the GS geometry or the S1 geometry. We then look if considering a larger oligomer would have an impact on the nature of the excited states. Here again we see little change in the properties of the excited states upon relaxation, and the electron and hole distance is around 6-7 Å.

Considering either the thermally accessible molecular geometries or the ones accessed upon relaxation of the molecule in the excited state, we could not find any evidence of a low energy excited state with a strong CT character.

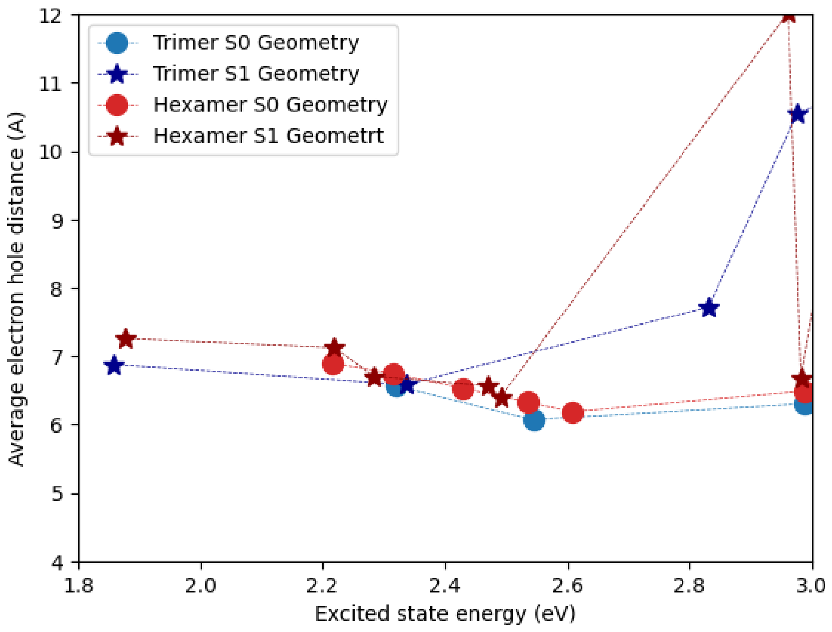

Figure S27: Excited state properties of IDTBT trimer and hexamer in the ground state optimized geometry or the optimized geometry of the first excited state. After the optimization of the molecules in the first or the ground state geometry, the vertical excited state energies and properties are computed. Here we see that upon relaxation the energy of the first excited state drops to 1.82 eV, and the average electron hole distance increases slightly by 0.1-0.2 A.

9.1.4. Impact of the solvent and the basis set and functional

Next we consider the impact of the presence of a solvent on the properties of the excited states inferred. In this case we only do the calculation on a trimer as it showed similar properties to that of the 6-mer. Figure S27 shows the impact of considering different polarized medium on the properties of the excited states of a trimer of IDT-BT. The presence of the solvent only slightly affects the energy of the excited state with little impact on the electron hole distance. This means that the presence of a solvent cannot justify the presence of low energy excited state with a strong charge transfer character.

We examined the influence of different density functional theory (DFT) methods on the prediction of excited state properties, specifically the relationship between excitation energies and electron-hole separation distances (RMSeh) for IDTBT systems (Figure S28). Three computational approaches were evaluated: B3LYP/6-31G* (a standard hybrid functional with a moderate basis set), CAM-B3LYP/cc-pVDZ (a range-separated hybrid functional with a correlation-consistent basis set), and ωB97XD/6-31G* (a range-separated functional with empirical dispersion correction). The results reveal significant method-

dependent variations in both excitation energies and electron-hole separation distances. B3LYP/6-31G* consistently predicts lower excitation energies (1.4-1.7 eV) but substantially larger RMSeh values (10-14 Å), suggesting overestimation of charge transfer character. In contrast, the range-separated functionals CAM-B3LYP and ωB97XD predict higher excitation energies (2.0-3.5 eV) with more compact electron-hole separations (5-10 Å), indicating better description of local excitonic states.

B3LYP suggest a higher charge transfer character for the excited state of IDT-BT, however they predict a considerably lower energy of the excited state, and the functional does not consider long range interactions which are important in describing a copolymer like IDT-BT.

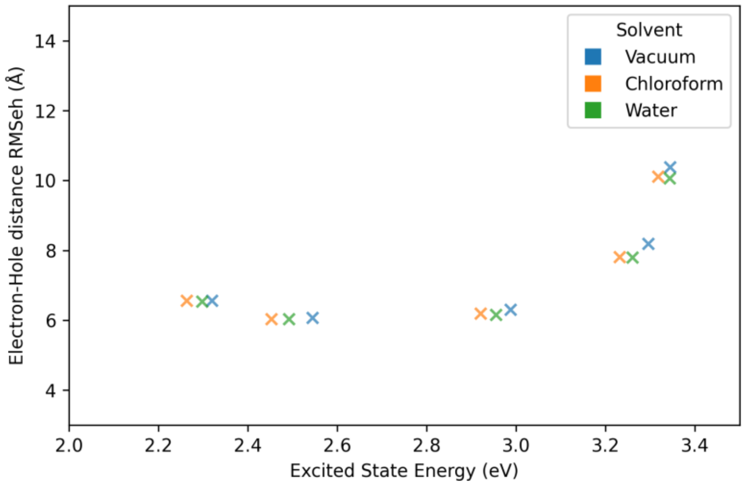

Figure S28 Excited state energy versus electron-hole separation distance for IDTBT 3-mer singlet states. TD-DFT calculations (CAM-B3LYP/cc-pVDZ) showing the relationship between excitation energy and spatial extent of electron-hole pairs in different solvents. Each point represents a singlet excited state in vacuum (blue), chloroform (orange), or water (green).

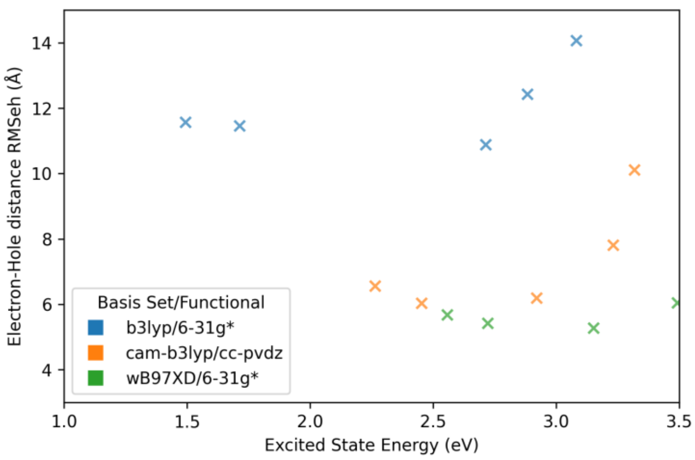

Figure S29 Comparison of density functional theory methods for excited state characterization of IDTBT systems. Correlation between excited state energy and electron-hole separation distance (RMSeh) calculated using three different DFT approaches: B3LYP/6-31G (blue), CAM-B3LYP/cc-pVDZ (orange), and ωB97XD/6-31G* (green). Each point represents a singlet excited state.*

9.2. Intermolecular Excited States

To investigate the potential of interchain pathways to polaron generation, we looked at the CT character of low-energy excited states in systems constituting pairs of 2mer chain segments of C8-IDTBT in close contact. We used extended tight binding molecular dynamics (xtb-md) to generate a statistical sample of conformations of the system. The initial conformation of the system was taken from an interchain contact structure present in a solid-state model of C16-IDTBT (see [13] for further details of how this model was produced), with sidechains shortened to C8. Notably, this initial conformation features the chains in a ‘crossing’ configuration, i.e., with their backbones angled perpendicular to one-another. Coker et al. showed that this crossing conformation is the predominant way by which C16-IDTBT chains make contact in the solid state.[13] Based on evidence from this same work, it is reasonable to assume that this arrangement will also be preferred for solution state aggregation and with C8 sidechains. The xtb-md simulation was performed at 298K with chloroform included as an implicit solvent. The simulation runtime was 100 ps, and snapshots were extracted every 1 ps, leading to an ensemble of 100 conformations. For the DFT calculations, the C8 sidechains on oligomers extracted from the xtb-md simulations for detailed study were shorted to methyl groups.

Since there may be small differences between the atomic positions predicted by xtb-md and DFT, each of the conformers was partially optimised (for 3 steps) using DFT at the b3lyp/6-31g level of theory. The partially optimised structures were then input into TDDFT simulations in which features of the first 10 triplet and first 10 singlet states were calculated, at the b3lyp/6-31g* level of theory. These initial DFT and TDDFT simulations were performed on the IDTBT structures in vacuum (see section 8.2.2 for the effect of a polarizable environment).

The post-TDDFT analysis code TheoDore was then applied to evaluate the CT character of the excited states. To quantify the degree of CT character, atoms within the systems are assigned to 'fragments'. In this case, a simple fragmentation scheme was applied in which the two chain segments were set as different fragments. The results of the TheoDore analysis are shown in Figure S29. The figure (a) shows the energies and oscillator strengths of transitions calculated from the ensemble of conformers. Each bar is coloured according to the CT character of that transition. Only singlet transitions are shown, since all triplets were found to have negligible oscillator strength.

The key result from this data can be seen in the cluster of red bars at the left-hand (lower energy) edge of the ensemble of transitions. This cluster indicates the presence of a low-energy excited state with significant CT character. Such a state may indicate support for polaron generation via an intermolecular pathway.

The visualisations shown in Figure S29 (b) can help clarify the nature of this low energy CT state. We can observe that the example transition marked by '*' leads to a significant build-up of electron density on one of the contacting backbone segments, while hole density increases on the opposing segment. This may be contrasted against the other highlighted transition, marked by '†'. In this case the transition shows more Frenkel-like character, with the change in electron and hole densities both largely confined to one of the two segments.

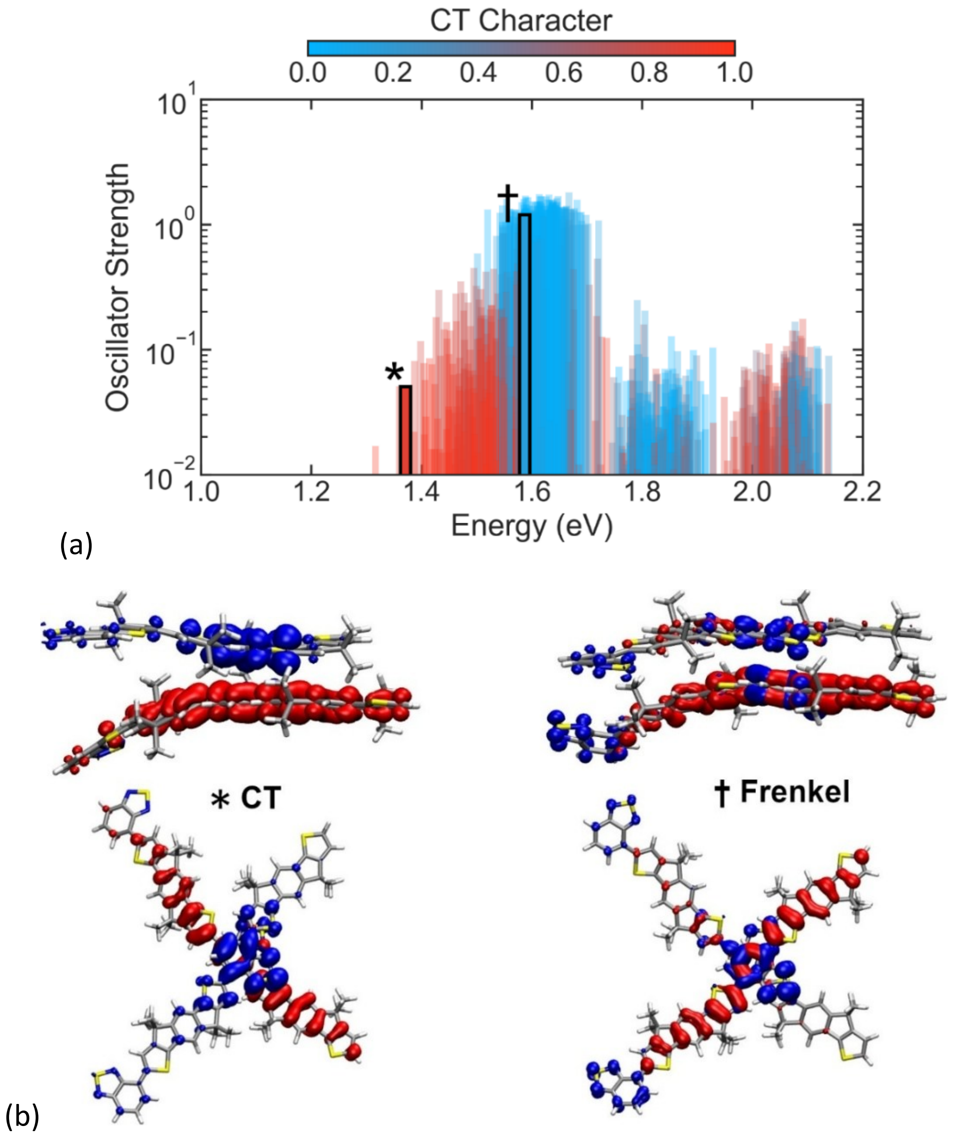

Figure S30: (a) Oscillator strength and quantified CT character of singlet excitations for a system of two IDTBT chain segments (2mers) in close backbone contact. Each semi-transparent bar represents a particular transition occurring for a particular snapshot (conformer) of the evolving system. Two example transitions have been highlighted by outlining in black. (b) Visualisation of the change in charge density associated with the two transitions highlighted on (a). The blue isosurface indicates an increase of electron density, while the red isosurface indicates a decrease in electron density, i.e., an increase in hole density. Two views (side-on and from above) are shown for each transition.

9.2.1. Impact of Basis set and Functional

To confirm the validity of the identified low energy CT state, we repeated the analysis procedure (partial optimisation with DFT, followed by TDDFT, followed by TheoDore analysis) with both DFT steps performed at different levels of theory. We compared the b3lyp and cam-

b3lyp functionals, as well as the 6-31g* and cc-pvdz basis sets. Here, only three snapshots were analysed, rather than the full ensemble.

The results of this analysis may be seen in Figure S30. While the effect of changing basis set appears to be minor, a significant change in the predicted behaviour was observed when switching to the cam-b3lyp functional. In contrast with the results from applying b3lyp, the lowest energy transitions now show little CT character. The observation of a lowest energy excited state with more Frenkel-like character is no longer consistent with polaron generation as occurring via an intermolecular pathway, due to the strong Coulomb attraction hindering charge separation. Thus, with respect to an intermolecular pathway to polaron generation, our results show significant functional dependence and are therefore inconclusive.

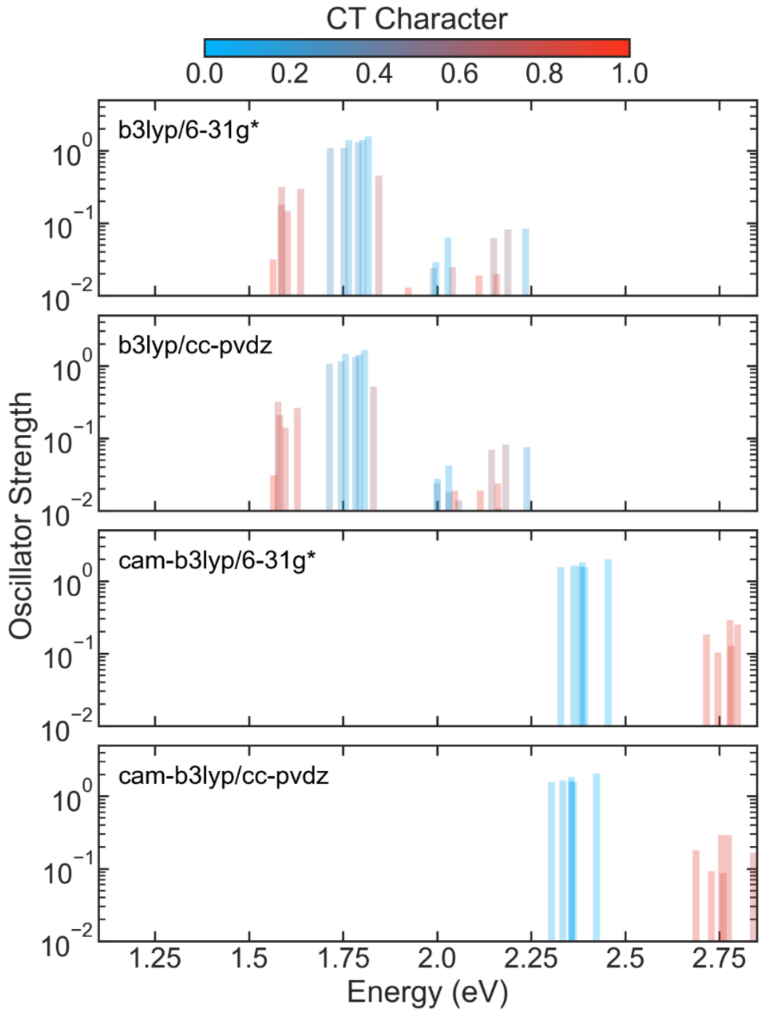

Figure S31: Oscillator strength and quantified CT character of singlet excitations for a system of two IDTBT chain segments (2mers) in close backbone contact as obtained using different levels of theory. Each semi-transparent bar represents a particular transition occurring for a particular snapshot (conformer) of the evolving system. Results are shown for three snapshots.

9.2.2. Impact of Solvent

To investigate the potential impact of including solvent on the nature of the interchain excited states, we repeated the analysis procedure (partial optimisation with DFT, followed by TDDFT, followed by TheoDore analysis) while including implicit solvent in the DFT and TDDFT steps. Both chloroform and water were tested as implicit solvent choices. For this analysis, only two levels of theory were tested (b3lyp/6-31g* and cam-b3lyp/cc-pvdz), based on our previous observation that the impact of changing the basis set alone was negligible. The same three snapshots were analysed as in the previous subsection.

As can be seen in Figures S31 and S32, including implicit solvent in the DFT and TDDFT steps of the analysis has the effect of shifting the lowest energy transitions to slightly higher energies (please note the different scaling of the x-axes compared to Figure S30). There is, however, relatively little effect on the CT character of the lowest energy transitions. Furthermore, there appears to be only a negligible difference between the results when using chloroform as the implicit solvent (Figure S31), compared to using water (Figure S32).

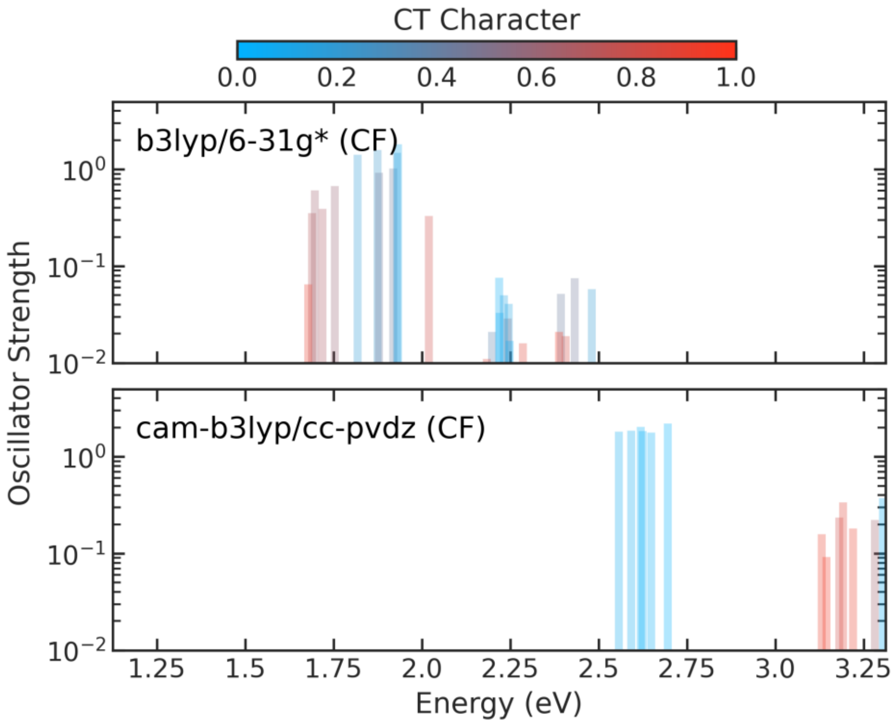

Figure S32: Oscillator strength and quantified CT character of singlet excitations for a system of two IDTBT chain segments (2mers) in close backbone contact as obtained using different levels of theory and implicit chloroform solvent. Each semi-transparent bar represents a particular transition occurring for a particular snapshot (conformer) of the evolving system.

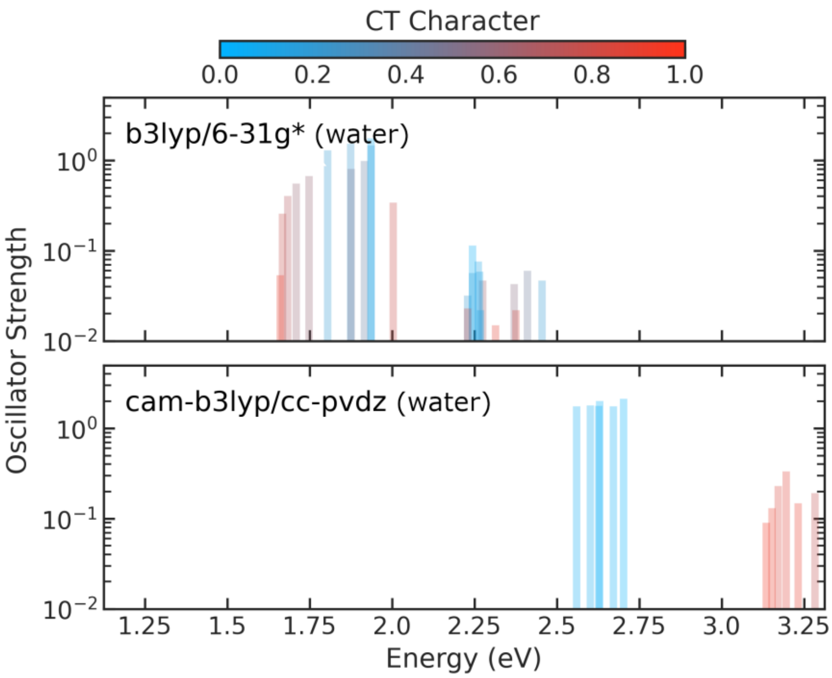

Figure S33: Oscillator strength and quantified CT character of singlet excitations for a system of two IDTBT chain segments (2mers) in close backbone contact as obtained using different levels of theory and implicit water solvent. Each semi-transparent bar represents a particular transition occurring for a particular snapshot (conformer) of the evolving system. Results are shown for three snapshots.

10. References

(1) Frisch, M. J.; Trucks, G. W.; Schlegel, H. B.; Scuseria, G. E.; Robb, M. A.; Cheeseman, J. R.; Scalmani, G.; Barone, V.; Petersson, G. A.; Nakatsuji, H.; Li, X.; Caricato, M.; Marenich, A. V.; Bloino, J.; Janesko, B. G.; Gomperts, R.; Mennucci, B.; Hratchian, H. P.; Ortiz, J. V.; Izmaylov, A. F.; Sonnenberg, J. L.; Williams; Ding, F.; Lipparini, F.; Egidi, F.; Goings, J.; Peng, B.; Petrone, A.; Henderson, T.; Ranasinghe, D.; Zakrzewski, V. G.; Gao, J.; Rega, N.; Zheng, G.; Liang, W.; Hada, M.; Ehara, M.; Toyota, K.; Fukuda, R.; Hasegawa, J.; Ishida, M.; Nakajima, T.; Honda, Y.; Kitao, O.; Nakai, H.; Vreven, T.; Throssell, K.; Montgomery Jr., J. A.; Peralta, J. E.; Ogliaro, F.; Bearpark, M. J.; Heyd, J. J.; Brothers, E. N.; Kudin, K. N.; Staroverov, V. N.; Keith, T. A.; Kobayashi, R.; Normand, J.; Raghavachari, K.; Rendell, A. P.; Burant, J. C.; Iyengar, S. S.; Tomasi, J.; Cossi, M.; Millam, J. M.; Klene, M.; Adamo, C.; Cammi, R.; Ochterski, J. W.; Martin, R. L.; Morokuma, K.; Farkas, O.; Foresman, J. B.; Fox, D. J. *Gaussian 16 Rev. C.01*; 2016.

(2) Lu, T.; Chen, F. Multiwfn: A multifunctional wavefunction analyzer. *Journal of Computational Chemistry* **2012**, *33* (5). DOI: 10.1002/jcc.22885

(3) Wadsworth, A.; Chen, H.; Thorley, K. J.; Cendra, C.; Nikolka, M.; Bristow, H.; Moser, M.; Salleo, A.; Anthopoulos, T. D.; Sirringhaus, H.; McCulloch, I. Modification of Indacenodithiophene-Based Polymers and Its Impact on Charge Carrier Mobility in Organic

Thin-Film Transistors. *Journal of the American Chemical Society* **2020**, *142* (2), 652-664. DOI: 10.1021/jacs.9b09374

(4) Coker, J. F.; Moro, S.; Gertsen, A. S.; Shi, X.; Pearce, D.; van der Schelling, M. P.; Xu, Y.; Zhang, W.; Andreasen, J. W.; Snyder, C. R.; Richter, L. J.; Bird, M. J.; McCulloch, I.; Costantini, G.; Frost, J. M.; Nelson, J. Perpendicular crossing chains enable high mobility in a noncrystalline conjugated polymer. *Proceedings of the National Academy of Sciences* **2024**, *121* (37). DOI: doi:10.1073/pnas.2403879121

(5) Venkateshvaran, D.; Nikolka, M.; Sadhanala, A.; Lemaur, V.; Zelazny, M.; Kepa, M.; Hurhangee, M.; Kronemeijer, A. J.; Pecunia, V.; Nasrallah, I.; Romanov, I.; Broch, K.; McCulloch, I.; Emin, D.; Olivier, Y.; Cornil, J.; Beljonne, D.; Sirringhaus, H.; Venkateshvaran, D.; Nikolka, M.; Sadhanala, A.; Lemaur, V.; Zelazny, M.; Kepa, M.; Hurhangee, M.; Kronemeijer, A. J.; Pecunia, V.; Nasrallah, I.; Romanov, I.; Broch, K.; McCulloch, I.; Emin, D.; Olivier, Y.; Cornil, J.; Beljonne, D.; Sirringhaus, H. Approaching disorder-free transport in high-mobility conjugated polymers. *Nature* **2014** *515* (7527), 384-388. DOI: 10.1038/nature13854

(6) Street, R. A.; Northrup, J. E.; Salleo, A. Transport in polycrystalline polymer thin-film transistors. *Physical Review B* **2005**, *71* (16). DOI: 10.1103/PhysRevB.71.165202

(7) Tanase, C.; Meijer, E. J.; Blom, P. W. M.; Leeuw, D. M. d. Local charge carrier mobility in disordered organic field-effect transistors. *Organic Electronics* **2003**, *4* (1). DOI: 10.1016/S1566-1199(03)00006-5

(8) Few, S.; Frost, J. M.; Kirkpatrick, J.; Nelson, J. Influence of Chemical Structure on the Charge Transfer State Spectrum of a Polymer:Fullerene Complex. *The Journal of Physical Chemistry C* **2014**, *118* (16), 8253-8261. DOI: 10.1021/jp412449n

(9) Plasser, F. Visualisation of Electronic Excited-State Correlation in Real Space. *ChemPhotoChem* **2019**, *3* (9), 702-706. DOI: 10.1002/cptc.201900014

(10) Souza, P. C. T.; Alessandri, R.; Barnoud, J.; Thallmair, S.; Faustino, I.; Grünewald, F.; Patmanidis, I.; Abdizadeh, H.; Bruininks, B. M. H.; Wassenaar, T. A.; Kroon, P. C.; Melcr, J.; Nieto, V.; Corradi, V.; Khan, H. M.; Domański, J.; Javanainen, M.; Martinez-Seara, H.; Reuter, N.; Best, R. B.; Vattulainen, I.; Monticelli, L.; Periole, X.; Tieleman, D. P.; de Vries, A. H.; Marrink, S. J.; Souza, P. C. T.; Alessandri, R.; Barnoud, J.; Thallmair, S.; Faustino, I.; Grünewald, F.; Patmanidis, I.; Abdizadeh, H.; Bruininks, B. M. H.; Wassenaar, T. A.; Kroon, P. C.; Melcr, J.; Nieto, V.; Corradi, V.; Khan, H. M.; Domański, J.; Javanainen, M.; Martinez-Seara, H.; Reuter, N.; Best, R. B.; Vattulainen, I.; Monticelli, L.; Periole, X.; Tieleman, D. P.; de Vries, A. H.; Marrink, S. J. Martini 3: a general purpose force field for coarse-grained molecular dynamics. *Nature Methods 2021 18:4* **2021**, *18* (4). DOI: 10.1038/s41592-021-01098-3

(11) Wassenaar, T. A.; Pluhackova, K.; Bockmann, R. A.; Marrink, S. J.; Tieleman, D. P. Going Backward: A Flexible Geometric Approach to Reverse Transformation from Coarse Grained to Atomistic Models. *J Chem Theory Comput* **2014**, *10* (2), 676-690. DOI: 10.1021/ct400617g

(12) William L. Jorgensen; David S. Maxwell, a.; Tirado-Rives, J. Development and Testing of the OPLS All-Atom Force Field on Conformational Energetics and Properties of Organic Liquids. *Journal of the American Chemical Society* **1996**, *118* (45). DOI: 10.1021/ja9621760

(13) Coker, J. F.; Moro, S.; Gertsen, A. S.; Shi, X.; Pearce, D.; van der Schelling, M. P.; Xu, Y.; Zhang, W.; Andreasen, J. W.; Snyder, C. R.; Richter, L. J.; Bird, M. J.; McCulloch, I.; Costantini, G.; Frost, J. M.; Nelson, J. Perpendicular crossing chains enable high mobility in a

noncrystalline conjugated polymer. *Proc Natl Acad Sci U S A* **2024**, *121* (37), e2403879121. DOI: 10.1073/pnas.2403879121
